# Expression of a Neuroendocrine Gene Signature in Gastric Tumor Cells from *CEA 424*-SV40 Large T Antigen-Transgenic Mice Depends on SV40 Large T Antigen

**DOI:** 10.1371/journal.pone.0029846

**Published:** 2012-01-13

**Authors:** Fritz Ihler, Elena Viviana Vetter, Jie Pan, Robert Kammerer, Svenja Debey-Pascher, Joachim L. Schultze, Wolfgang Zimmermann, Georg Enders

**Affiliations:** 1 Walter Brendel Centre of Experimental Medicine, University of Munich, Munich, Germany; 2 IFBLMU - Integrated Center for Research and Treatment of Vertigo, Balance and Ocular Motor Disorders, University of Munich Hospital, Munich, Germany; 3 Department of ENT - Head and Neck Surgery, University of Göttingen Hospital, Göttingen, Germany; 4 Tumor Immunology Laboratory, LIFE Center, University of Munich Hospital, Munich, Germany; 5 Institute of Immunology, Friedrich Loeffler Institute, Tübingen, Germany; 6 Genomics & Immunoregulation, Life & Medical Sciences Institute (LIMES), University of Bonn, Bonn, Germany; University of Missouri-Columbia, United States of America

## Abstract

**Background:**

A large fraction of murine tumors induced by transgenic expression of SV40 large T antigen (SV40 TAg) exhibits a neuroendocrine phenotype. It is unclear whether SV40 TAg induces the neuroendocrine phenotype by preferential transformation of progenitor cells committed to the neuroendocrine lineage or by transcriptional activation of neuroendocrine genes.

**Methodology/Principal Findings:**

To address this question we analyzed *CEA424*-SV40 TAg-transgenic mice that develop spontaneous tumors in the antral stomach region. Immunohistology revealed expression of the neuroendocrine marker chromogranin A in tumor cells. By ELISA an 18-fold higher level of serotonin could be detected in the blood of tumor-bearing mice in comparison to nontransgenic littermates. Transcriptome analyses of antral tumors combined with gene set enrichment analysis showed significant enrichment of genes considered relevant for human neuroendocrine tumor biology. This neuroendocrine gene signature was also expressed in 424GC, a cell line derived from a *CEA424*-SV40 TAg tumor, indicating that the tumor cells exhibit a similar neuroendocrine phenotype also *in vitro*. Treatment of 424GC cells with SV40 TAg-specific siRNA downregulated expression of the neuroendocrine gene signature.

**Conclusions/Significance:**

SV40 TAg thus appears to directly induce a neuroendocrine gene signature in gastric carcinomas of *CEA424*-SV40 TAg-transgenic mice. This might explain the high incidence of neuroendocrine tumors in other murine SV40 TAg tumor models. Since the oncogenic effect of SV40 TAg is caused by inactivation of the tumor suppressor proteins p53 and RB1 and loss of function of these proteins is commonly observed in human neuroendocrine tumors, a similar mechanism might cause neuroendocrine phenotypes in human tumors.

## Introduction

A number of different strategies have been adopted to create transgenic murine tumor models which mirror human malignant disease. One strategy includes the use of oncogenes the expression of which is driven by tissue-specific promoters. Simian virus 40 large T antigen (SV40 TAg) is commonly employed as an oncogene to reliably elicit tumors in transgenic mice due to its capability to simultaneously inactivate p53 and retinoblastoma (RB) proteins (pRB, p107, p130), prominent cellular tumor suppressors. Inactivation of the RB proteins leads to loss of suppression of a family of E2F transcription factors which in turn induce expression of cell cycle-promoting genes. In addition, inactivation of p53 switches off genes which encode apoptosis-inducing proteins thus allowing quiescent cells to re-enter S-phase and to escape apoptosis [1]. In that way a large panel of transgenic mouse strains has been established which develop tumors in a wide spectrum of organs, e.g. colon, stomach, prostate, pancreas and lung [2], [3]. Surprisingly, the majority of SV40 TAg-induced tumors exhibit a neuroendocrine phenotype [4], [5], [6], [7], [8], [9]. In contrast, in humans neuroendocrine tumors represent only a minor fraction accounting for some 1–3% of all tumors [10]. Neuroendocrine tumors are supposed to be of neuroectodermal origin and possess properties typical of neuroendocrine cells, like containing secretory granules, producing neuroendocrine factors including chromogranin, synaptophysin and specific hormones [11].

We have previously established a transgenic mouse strain which expresses SV40 TAg under the control of a human carcinoembryonic antigen (*CEA*) minimal promoter (*CEA424*-SV40 TAg; [12]). Those mice develop with 100% penetrance antral stomach tumors at an early age and die from the tumor at an age of about 115 days. Based on the expression of the epithelial marker EpCAM in derived tumor cell lines and transgenic human *CEA* which is widely expressed in mucus-producing epithelial cells and adenocarcinomas we tentatively classified these tumors as adenocarcinomas [12], [13], [14]. The undifferentiated nature of *CEA424*-SV40 TAg tumors and the high incidence of neuroendocrine tumors among SV40 TAg-induced tumors, however, prompted us to reinvestigate the gastric tumors of *CEA424*-SV40 TAg mice. Indeed, transcriptome, immunohistological and electron microscopic analysis revealed typical neuroendocrine features of these tumors.

From this the question arises whether SV40 TAg is directly responsible for expression of the genes encoding the neuroendocrine proteins. To answer this question, we downregulated by siRNA the expression of SV40 TAg in 424GC tumor cells, a cell line which was generated from a primary tumor of *CEA424*-SV40 TAg-transgenic mice [13] and determined the transcriptome of treated and non-treated cells. As expected, we observed downregulation of genes which are activated by E2F transcription factors. Upon silencing of SV40 TAg mRNA, the SV40 TAg can no longer inhibit the RB protein, itself a repressor of E2F proteins. Surprisingly, gene set enrichment analysis (GSEA) revealed that a group of genes characteristically expressed in human neuroendocrine tumors were also significantly downregulated. These findings might explain the often observed association of SV40 TAg transgenesis and neuroendocrine tumor formation.

## Results

### *CEA424*-SV40 TAg gastric tumors exhibit a neuroendocrine phenotype

**Figure 1:**
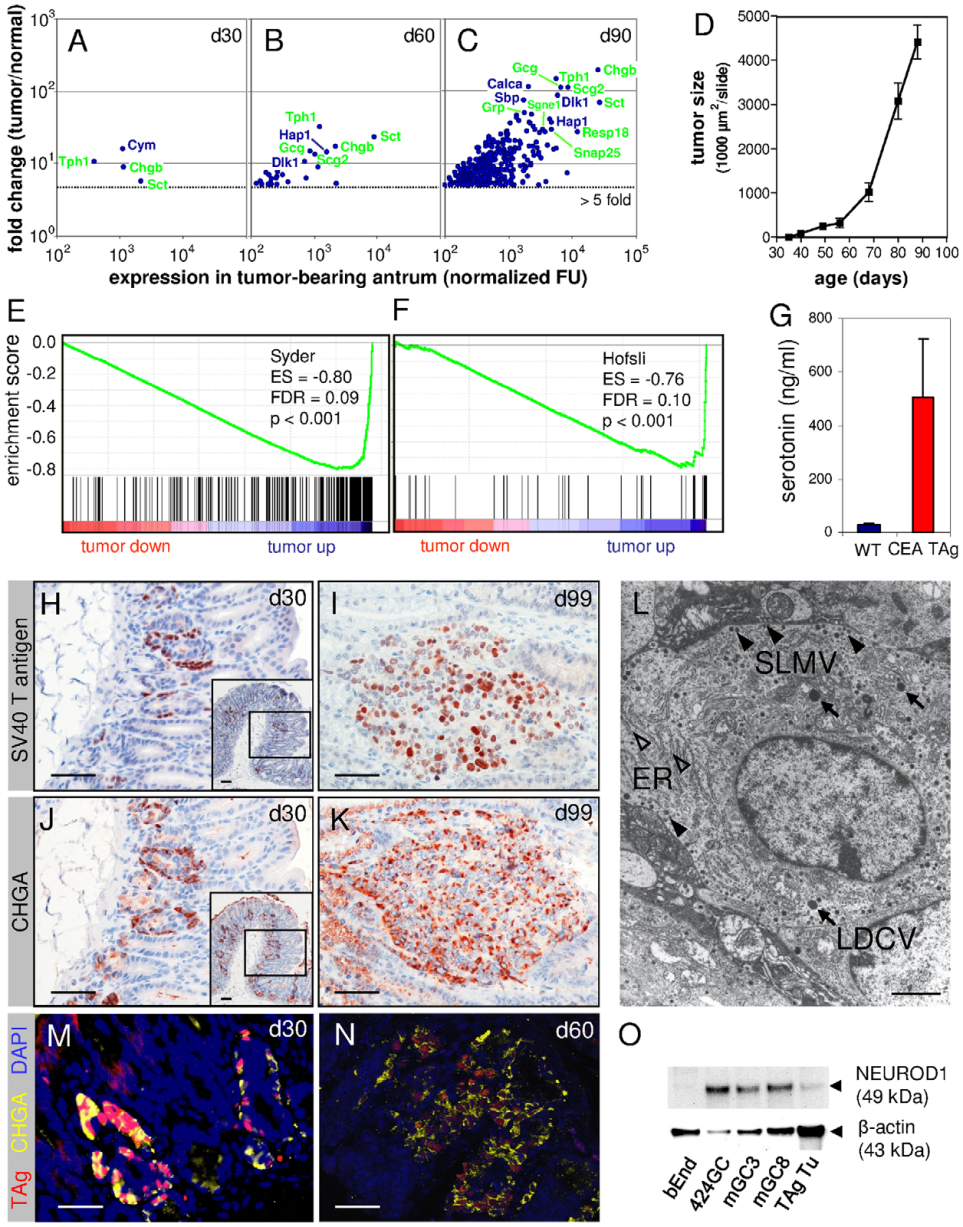
*CEA424*-SV40 TAg gastric tumors exhibit a neuroendocrine phenotype. Transcriptome analysis of RNA from tumor-bearing regions of the antrum from 30- (A), 60- (B) and 90-day-old *CEA424*-SV40 TAg-transgenic mice (C) and non-transgenic mice revealed predominant enrichment of genes typically expressed in neuroendocrine tissues (symbols shown in green). The means of the expression levels of genes preferentially expressed in tumor-bearing stomach tissues were plotted against their expression ratio tumor/normal stomach (genes with fold change >5-fold are shown; n = 3). The most strongly upregulated and most highly expressed genes in the samples are identified by gene symbols. These were statistically significant for day 90 (p<0.05). Note the strong increase of upregulated genes with age which reflects the exponential tumor growth between d30 and d90 in *CEA424*-SV40 TAg transgenic mice (D). (E, F) Gene set enrichment analysis (GSEA) of the d90 tumor ranked gene list using the neuroendocrine signature genes from murine *Atp4b*-SV40 TAg gastric tumors ([4]; E) and from human neuroendocrine tumors ([15]; F) comprising 305 identifiable genes out of 399 genes and 39 identifiable genes out of 52 genes, respectively. ES, enrichment score; FDR, false discovery rate; tumor up/down, signature gene sets up- or downregulated in tumors. (G) Tumor-bearing transgenic mice exhibit elevated serum serotonin levels. Serum from wildtype (n = 6 pooled; age: 130 d) and *CEA424*-SV40 TAg mice (n = 6; age: 95–110 d) were analyzed by ELISA. (H–K) Gastric tumor cells express both SV40 TAg (brown nuclear staining; H, I) and the neuroendocrine marker chromogranin A (CHGA; brown cytoplasmic staining; J, K). Parallel sections of formalin-fixed paraffin-embedded gastric tumors from 30- and 99-day-old mice were used for immunohistological staining. The tumors were localized in the antrum and duodenum, respectively. Latter tumors were commonly observed in mice older than 90 days and are supposed to have originated from pyloric tumors through invasive growth or metastatic spread. (M, N) Double-staining with fluorescently labeled anti-SV40 TAg and CHGA of gastric tumor-bearing tissue from 30- and 60-day-old mice. Note most of the cells with SV40 TAg-positive nuclei (red color) express CHGA (yellow color). The nuclei are visualized by DAPI (blue color). Bars: 50 µm. (L) Transmission EM of a typical tumor cell in a tumor from a 90-day-old *CEA424*-SV40 TAg mouse. Note the numerous electron-dense secretory granules (LDCV, large dense core vesicles, arrows; SLMV, small synaptic-like microvesicles arrowheads) and extended mesh of rough endoplasmic reticulum (ER; open arrowheads) typical of neuroendocrine and rapidly growing cells, respectively. Original magnification: ×15.300 in (L). Bars: 50 µm in H-K; 2 µm in L. (O) Western blot analysis of NEUROD1 expression in *CEA424*-SV40 TAg tumor-derived cell lines (424GC, GC3, GC8) and in tumors from 85-day-old mice. The endothelioma cell line served as a negative control, detection of β-actin as loading control.

In order to reinvestigate the previous classification of *CEA424*-SV40 TAg tumors as adenocarcinomas [12] we performed genome-wide expression analyses with RNA from the antral region from normal and tumor-bearing mice. Stomachs from 30-, 60- and 90-day-old mice were analyzed. At day 30 (d30), *CEA424*-SV40 TAg-transgenic mice exhibit small multifocal tumor lesions in the pyloric region of the antrum (Figure 1 H, inset) which grow exponentially leading to extended tumors at an age of 90 days which infiltrate into the duodenum and cause death within a further 2–3 weeks probably by pyloric stenosis (Figure 1 D). Four, 27 and 338 probe sets/genes were found to be more than 5-fold expressed in tumor-bearing antral regions from 30, 60 and 90 day old transgenic mice, respectively, when compared to antra from normal littermates (Figure 1 A–C). 272 of the genes upregulated >5-fold in the stomachs of 90 day old transgenic mice were overexpressed at a significance level of p<0.05 (Table S1). Interestingly, a substantial fraction of the most highly expressed and most strongly upregulated genes comprised genes characteristic for the neuroendocrine lineage, e.g. genes encoding chromogranin B (*Chgb*), secretin (*Sct*), glucagon (*Gcg*), secretogranin II (*Scg2*) and tryptophan hydroxylase (*Tph1*) (Figure 1 A–C; Table S2). This finding was substantiated using gene set enrichment analyses (GSEA) by comparing the d90 tumor versus normal tissue ranked gene list with a list of 399 genes preferentially expressed after transdifferentiation of ATP4B-expressing gastric preparietal progenitor cells (pPC), without neuroendocrine features, into locally invasive or metastatic neuroendocrine tumor cells (iGC/mGC) in gastric tumors of *Atp4b* promoter-SV40 TAg-transgenic mice (Table 5 in [4]). This list of mouse genes overlaps with genes that were found to be typically expressed in human neuroendocrine lung cancers [4]. In addition, a list of 52 genes assembled by Hofsli et al. (Table 3 in [15]) which are characteristically expressed in human neuroendocrine tumors and are thought to play a role in neuroendocrine tumor biology was used to determine selective enrichment of “neuroendocrine tumor genes” in *CEA424*-SV40 TAg tumors. Most significantly, both sets of genes were highly enriched in the group of genes upregulated in tumors of 90-day-old mice exhibiting low false discovery rates (FDR) at a high significance level (p<0.001; Figure 1 E, F; Table 1).

**Table 1:** Gene set enrichment analyses (GSEA) of transcriptomes of d90 *CEA424*-SV40 TAg tumors and of 424GC cells after SV40 TAg siRNA treatment using neuroendocrine and E2F target gene signatures.

Sample	Gene set	Number of applicable gene sets (total)	Enrichment score (ES)	p value^1^	False discovery rate (FDR)
d90 gastric tumors	Syder^2^	305 (399)	−0.800	**<0.001**	0.090
424GC	Syder^2^	256 (399)	−0.443	0.481	0.657
d90 gastric tumors	Syder (transcription factors)^3^	25 (32)	−0.860	**<0.001**	0.112
424GC	Syder (transcription factors)^3^	25 (32)	−0.657	0.402	0.585
d90 gastric tumors	Hofsli^4^	39 (52)	−0.757	**<0.001**	0.120
424GC	Hofsli^4^	36 (52)	0.858	**<0.001**	0.125
d90 gastric tumors	Schaffer^5^	19 (23)	−0.898	**<0.001**	0.081
424GC	Schaffer^5^	23 (23)	0.853	**<0.001**	0.180
d90 gastric tumors	Cantalupo^6^ (E2F)	38 (42)	−0.689	0.184	0.261
424GC	Cantalupo^6^ (E2F)	39 (42)	0.883	**<0.001**	0.147

1 p values<0.001 in bold;

2 Syder et al. 2004, Table S5 [4];

3 Syder et al. 2004, Table S7 [4];

4 Hofsli 2006, Table 3 [15];

5 Schaffer et al. 2010, Figure 4a [30];

6 Cantalupo et al. 2009, Table S5 [19].

In order to see whether overexpression of *Tph1* in d90 tumor tissue (Figure 1 C; Table S1) translates into higher serotonin blood levels in tumor-bearing mice, we measured serotonin serum concentrations in wildtype and *CEA424*-SV40 TAg-transgenic mice using ELISA. Tryptophan hydroxylase catalyzes the rate-limiting step in the conversion of L-tryptophan to serotonin with 5-hydroxy-L-tryptophan as an intermediate product. In sera of transgenic tumor-bearing mice 18-fold higher serotonin levels were found (Figure 1 G).

Expression of *Chga* mRNA was 15-fold upregulated in d90 tumors in comparison to normal antrum (Table S1). Upregulation could be confirmed at the protein level by immunohistology. Most of the tumor cells which were identified by nuclear expression of SV40 TAg in parallel tissue sections of 30- and 90-day-old *CEA424*-SV40 TAg-transgenic mice (Figure 1 H, I) strongly expressed chromogranin A (Figure 1 J, K). Double-labeling with fluorescent antibodies corroborated coexpression of SV40 TAg and CHGA in a large fraction of SV40 TAg-positive tumor cells in d30 and d60 tumors (Figure 1M, N). Furthermore, electron microscopy (Figure 1 L) revealed numerous electron-dense secretory granules in the cytoplasm of tumor cells which are characteristically observed in neuroendocrine cells [4]. Two different size classes could be discriminated: the small probably represent so called synaptic-like microvesicles (SLMV) and the larger granules large dense core vesicles (LDCV) which are known to contain synaptophysin and chromogranin A, respectively [16].

To check whether SV40 TAg is also expressed in gastric epithelial progenitors, we costained gastric tissue sections from 30-, 60- and 90-day-old *CEA424*-SV40 TAg mice with an acid mucin-detecting dye (Alcian blue) and anti-SV40 TAg antibodies. No Alcian blue staining was observed in SV40 TAg-positive cells. Less than 5% of TAg-positive cells were found in close vicinity of Alcian blue-stained cells in 30-day-old mice (Figure S1).

In order to identify regulatory proteins which might be responsible for the development of the neuroendocrine phenotype we looked at the expression of a subset of 32 genes from the 399 member gene list compiled by Syder et al. (Table 7 in [4]). These genes encode transcription factors and DNA-binding proteins. Out of this list, 31 could be identified in our probe sets. Twenty-three out of these 31 genes were clearly expressed (>100 RFU). Transcripts of 16 of these genes (52%) were significantly (p<0.05) enriched in the d90 tumors compared to normal antral tissues (Table S3). Notably, among the most highly enriched transcripts were those of *Nkx2.2* and *Neurod1* that encode transcription factors known to be involved in the differentiation of the neuroendocrine lineage of gastrointestinal tissues [17]. NEUROD1 protein could be detected by Western blot in tumors from 85-day-old mice as well as in cell lines established from *CEA424*-SV40 TAg gastric tumors (Figure 1 O). In contrast, suppressors of the enteroendocrine lineage, HES1 and GFI1 which inhibit the activators of the enteroendocrine lineage ATOH1 and NEUROG3, respectively, are either not expressed (*Gfi1*) or depleted (*Hes1*; 1.5-fold; data not shown). mRNAs of transcription factors genes which support the formation of other intestinal lineages, like *Sox9* (Paneth cells), *Klf4* (goblet cells) and *Elf3* (goblet cells and enterocytes) are depleted in d90 tumors compared to normal antrum (data not shown). Interestingly, the gene encoding the transcription factor ETV1 which has recently been shown to represent a master regulator in gastrointestinal stromal (GIST) tumors is strongly expressed and its transcripts are more than 17-fold enriched in d90 tumors (Table S1, Table S3). GIST tumors are thought to be derived from intestinal interstitial cells of Cajal (ICC), a neuronal cell lineage [18].

### The *CEA424*-SV40 TAg gastric tumor-derived cell line 424GC transcriptome reflects the neuroendocrine phenotype of the parental tumor

**Figure 2:**
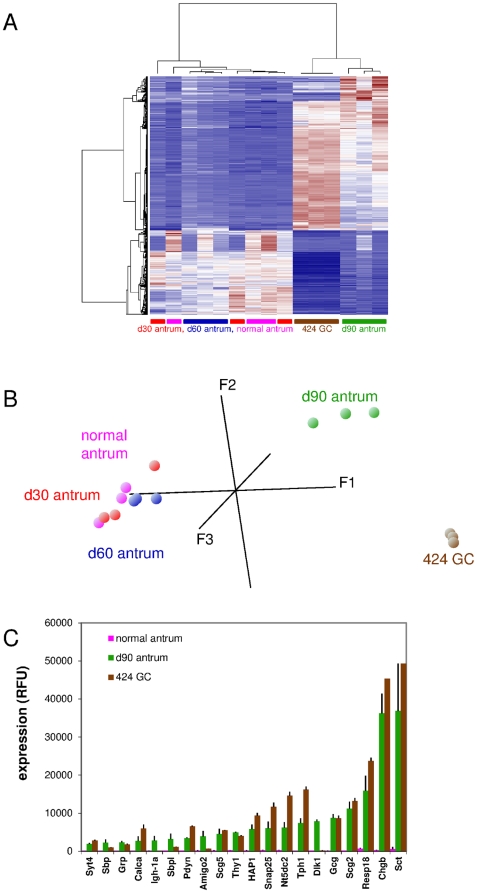
The *CEA424*-SV40 TAg gastric tumor-derived cell line 424GC transcriptome reflects the neuroendocrine phenotype of the parental tumor. Transcriptome analysis of RNA from tumor-bearing regions of the antrum from 30- (red), 60- (blue) and 90-day-old mice (green) and d90 non-transgenic mice (pink) as well as from 424GC cells (brown) were performed and the data sets were subjected to hierarchical clustering and principal component analyses (n = 3 for all samples). The heatmap shows that tumors at day 90 and 424GC show the most closely related expression pattern (A). Note that in PCA, data points of 424GC cells and that of d90 antra exhibit coordinates which are substantially different from that of all other samples (B). The expression levels (mean of 3 samples and standard deviation) of selected genes (expression ratio d90 antra/normal antra >15-fold) were compared to that observed for 424GC cells and normal stomach. If a gene was represented by more than one probe set the one which displayed the highest fold change value (expression d90 versus normal antra) was chosen (C). Note high expression of genes characteristic for the neuroendocrine lineage in both d90 antra and 424GC cells (e.g. tryptophan hydroxylase, *Tph1*; chromogranin B, *Chgb*; secretin, *Sct*).

A number of cell lines have been derived from primary gastric tumors of *CEA424*-SV40 TAg-transgenic mice [13]. Previous analyses of these cell lines were not aimed at the characterization of their potential neuroendocrine phenotype. Therefore, we compared the transcriptome of 424GC cells with that of the antral regions of 30-, 60-, 90-day-old *CEA424*-SV40 TAg-transgenic as well as of non-transgenic mice by hierarchical clustering and principal component (PCA) analysis. As expected, the antral stomach regions from normal, 30-day-and 60-day-old transgenic mice which have a very small tumor load (Figure 1 D) cluster together whereas d90 tumors differ substantially from that of normal tissue and are more similar to 424GC tumor cells (Figure 2 A). Similar results were obtained when performing PCA (Figure 2 B). In PCA the expression differences of various samples are condensed into one data point and displayed in a three dimensional coordinate system. Samples which exhibit similar expression pattern are found in close proximity. The cluster heatmap reveals several genes with high expression in d90 tumors and 424GC tumor cells in comparison to all other samples. The relationship of d90 tumors and 424GC transcriptomes was further exemplified by comparing the expression levels of genes which were more than 15-fold enriched in d90 tumors in comparison to normal antrum (Figure 2 C). Most strikingly, high expression at a similar level of genes encoding characteristic neuroendocrine lineage markers, like secretin (*Sct*), chromogranin B (*Chgb*), tryptophan hydroxylase 1 (*Tph1*), regulated endocrine-specific protein of 18 kDa (*Resp18*), synaptosomal associated 25 kDa protein (*Snap25*), secretogranin II (*Scg2*) and glucagon (*Gcg*), was found both in d90 tumors and 424GC cells.

### SV40 T antigen-specific siRNA downregulates expression of neuroendocrine genes in *CEA424*-SV40 TAg gastric cancer cell line

**Figure 3:**
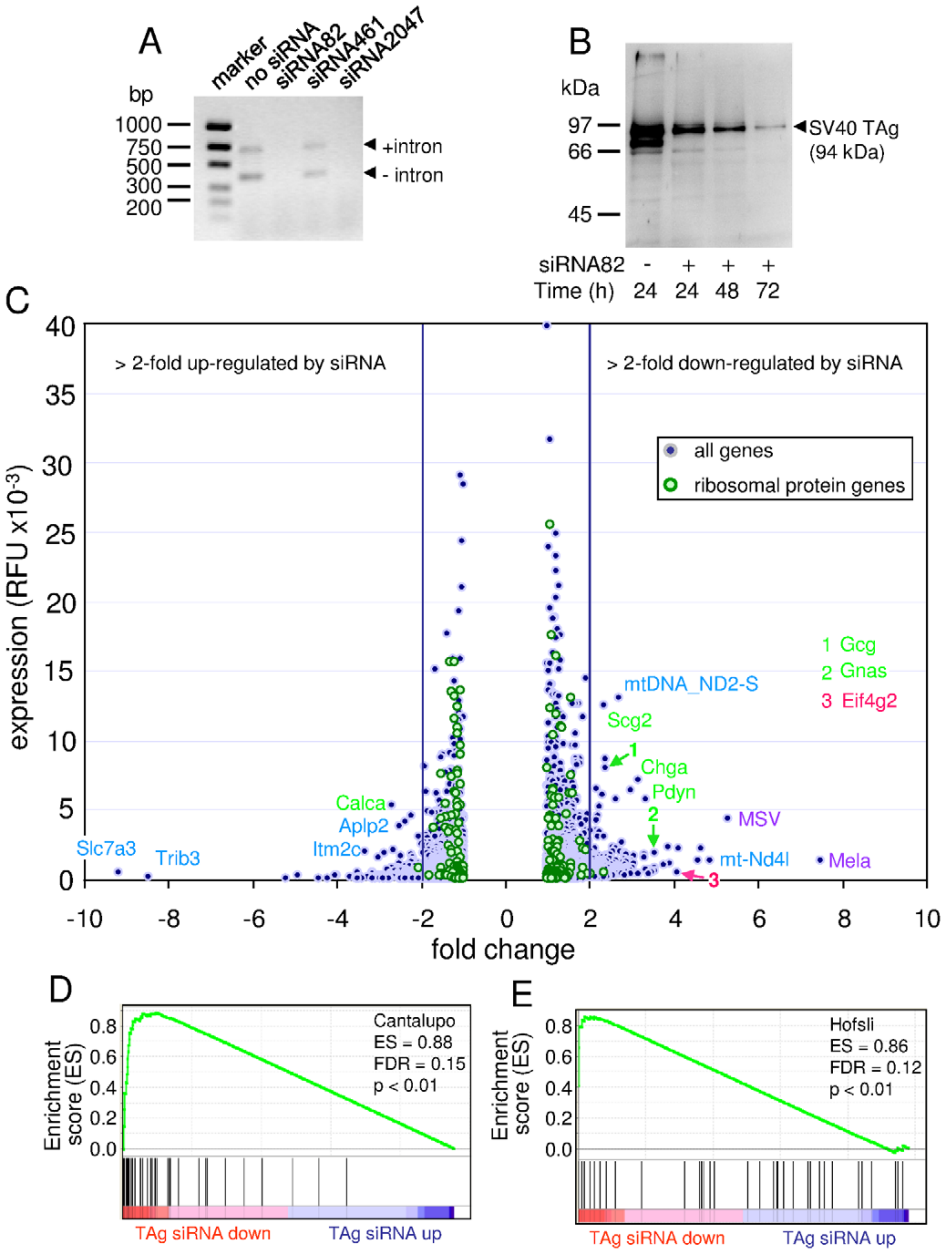
SV40 T antigen-specific siRNA downregulates expression of neuroendocrine genes in the *CEA424*-SV40 TAg gastric cancer cell line 424GC. Three SV40 TAg-targeting siRNAs were evaluated in 424GC cells. siRNA82 and siRNA2047 completely abrogated SV40 T antigen mRNA expression analyzed 72 h after transient transfection by RT-PCR (A) and Western blot analysis (siRNA82 only; B). The two PCR products represent both non-spliced and spliced SV40 TAg RNA. The identity of the 70–80 kDa band in the untreated cell sample is unclear. 72 h after addition of siRNA82 the transcriptome of 424GC cells was analyzed and compared to that of untreated cells. The expression levels of genes up- (negative ratios) or downregulated (positive ratios) by SV40 T antigen siRNA treatment were plotted against their expression ratio (C). The most strongly up- and downregulated and most highly expressed genes in the samples are identified by gene symbols and color codes (green, neuroendocrine; red, cell cycle/proliferation; purple, retroviral; blue, other genes). For comparison, the expression levels and fold chance values of all ribosomal protein genes were plotted. Note their low fluctuation of expression change (maximally 2-fold). The mean fold change (transcript level with/transcript level without siRNA treatment) for 244 probe sets was 1.07±0.25. The control experiment was performed trice (n = 3), the siRNA experiment twice (n = 2). GSEA of the SV40 TAg siRNA ranked gene list from treated and untreated 424GC cells using a neuroendocrine signature of 39 genes from human tumors as in Figure 1 ([15]; D) and 36 E2F target genes ([19]; E). ES, enrichment score; FDR, false discovery rate; TAg siRNA up/down, signature gene sets up- or downregulated in TAg siRNA-treated cells.

Because murine tumors induced by transgenic expression of SV40 TAg often exhibit a neuroendocrine phenotype we reasoned that knock-down of SV40 TAg by siRNA in SV40 TAg-positive neuroendocrine tumor cells could selectively downregulate expression of neuroendocrine genes. To this end we designed three different SV40 TAg-specific siRNAs and tested their ability to diminish SV40 TAg mRNA expression 72 h after transfection of 424GC cells. Both siRNA82 and siRNA2047 abrogated SV40 TAg mRNA expression completely as judged by reverse transcription polymerase chain reaction analysis (Figure 3 A). The silencing potency of siRNA82 was further analyzed by Western blot analysis. Seventy-two hours after siRNA transfection only marginal amounts of SV40 TAg protein could be detected (Figure 3 B).

We then analyzed the transcriptome of 424GC cells treated for 72 h with siRNA82 using oligonucleotide microarrays which included probe sets for SV40 TAg mRNA and compared it to that of untreated cells. The siRNA treatment led to a reduction of the level of SV40 TAg mRNA by 56% (p<0.01). The expression of genes encoding ribosomal proteins which we assumed to be unaffected by siRNA82 treatment varied maximally ∼2-fold (up or down; mean fc_with/without siRNA_ = 1.07±0.25) between the two samples (Figure 3 C green dots). Therefore, we considered a >2-fold change in mRNA levels as biologically relevant. SiRNA treatment of 424GC cells downregulated the expression of 269 genes/gene sets >2-fold, while 238 genes/gene sets showed a >2-fold upregulation (Figure 3 C; Table S4).

Not surprisingly, among the genes that were most strongly downregulated by treatment with SV40 TAg-specific siRNA (2.8–4.1-fold), were those that were frequently found to be involved in proliferation and cell cycle progression such as *Eif4g2* (eukaryotic translation initiation factor 4, gamma 2), *Ccnb1* (cyclin B1), *Pcna* (proliferating cell nuclear antigen) and *Mki67* (antigen identified by monoclonal antibody Ki67) (Table S4). Concomitantly, genes encoding cell cycle inhibitors and apoptosis-promoting proteins were upregulated (2.2–2.5-fold), among them *Cdkn2b* (cyclin-dependent kinase inhibitor 2B), *Pdcd4* (programmed cell death 4) and *Bax* (Bcl2-associated X protein) (Figure 3 C; Table S4). Many of the downregulated genes represent target genes of E2F transcription factors which are activated through the silencing of the RB protein by SV40 TAg [1]. Indeed, GSEA analysis of the ranked gene list obtained from siRNA-treated and untreated cells using a set of E2F target genes which was compiled by Cantalupo et al. [19] revealed a highly significant enrichment (p<0.01) of E2F target genes in the downregulated fraction (Figure 3 D; Table 1).

Notably, we observed also downregulation by SV40 TAg siRNA of genes encoding neuroendocrine markers (Figure 3 C; Table S4) like *Gnas* (3.5-fold), *Pdyn* (prodynorphin) (3.3-fold), *Chga* (3-fold), *Gcg* (2.4-fold) as well as *Scg2* and *Sst* (2.3-fold each). No neuroendocrine marker genes were significantly upregulated by SV40 TAg siRNA with the exception of *Calca* (2.7-fold) (calcitonin/calcitonin-related polypeptide alpha) (Figure 3 C; Table S4). These findings were corroborated by GSEA analyses which revealed significant enrichment (p<0.01) of genes selectively expressed in human endocrine tumors (Figure 3 E; Table 1) [15].

Furthermore, the highly expressed gene *Hey1* (*hairy/enhancer-of-split-related with YRPW motif*) which induces neuronal differentiation in the brain and represents a *bona fide* E2F target gene [20] was significantly transcriptionally downregulated (2-fold; p<0.01) in 424GC cells by TAg-specific siRNA (data not shown). On the other hand, none of the transcription factor genes which regulate the neuroendocrine lineage like *Nkx2.2* and *Neurod1* was significantly downregulated by TAg siRNA (data not shown).

To our surprise, the most strongly downregulated genes represented endogenous retroviral genes (*Mela, MSV, Abl-MLV*; Figure 3 C; Table S4). Regulation of endogenous retroviruses by SV40 TAg has been noted before and might be explained by direct transcriptional activation by TAg of promoter elements within retroviral long terminal repeats [21].

## Discussion

The SV40 TAg has been used for the induction of numerous organ-specific cancer models in transgenic mice, through control of its expression by a large variety of cell type-specific promoters [2]. Surprisingly, a large proportion of the published models (∼70%) express markers that are typically found in neuroendocrine tumors (Table S5, Supporting References S1), like peptide hormones (gastrin, gastrin-releasing peptide, somatostatin, glucagon), proteins stored in specific secretory granules (chromogranin A and B, synaptophysin) and neuroamines (serotonin, dopamine). Some of these substances are responsible for often severe symptoms such as flushing, diarrhea and dermatitis noticed in patients with certain neuroendocrine tumors [10]. In the model system presented here, 424 bp of the human *CEACAM5/CEA* promoter are used to drive the expression of the SV40 TAg antigen. Tumors develop in 100% of the animals in the antrum of the stomach [12] which were first tentatively identified as adenocarcinomas due to the expression of EpCAM and transgenic *CEA* [12], [13], [14]. In this study, immunohistological detection of chromogranin A and B, presence of high levels of serotonin in the blood as well electron microscopic analysis clearly demonstrated a neuroendocrine phenotype of gastric tumors of *CEA424*-SV40 TAg mice (Figure 1 and data not shown). In addition, transcriptome analyses of normal and tumor-bearing antral tissue revealed the enrichment of typical gene signatures found in human or murine neuroendocrine tumors. For example, 16 out of 42 genes overexpressed ≥20-fold in the antrum of 90 day old transgenic mice encode typical (neuro)endocrine proteins (CHGB, SCT, SCG2, TPH1, GCG, DLK1, CALCA, RESP18, SNAP25, GRP). Thus the gastric tumors of these mice exhibit a number of features that might be helpful to study the role of secreted substances (like serotonin) in NET syndrome formation as well as to develop new therapeutic strategies for the treatment of gastric neuroendocrine tumors.

What is the reason for the apparent overrepresentation of neuroendocrine tumors in SV40 TAg-transgenic mice? One possibility could be that TAg preferentially transforms neuroendocrine progenitor cells. However, this explanation does not apply in general and appears to be rather unlikely because there are SV40 TAg mouse tumor models with initial TAg expression in non-neuroendocrine epithelial cells from which neuroendocrine tumors develop by transdifferentiation [4], [9]. Syder et al. directed expression of TAg to acid-producing parietal cell progenitors of the stomach using the parietal cell-specific *Atp4b* promoter. Next to small ATP4B- and TAg-positive tumors, ATP4B-negative invasive neuroendocrine tumors develop after a long latency period of more than 300 days. Similarly, Czeh et al. observed both well differentiated adenocarcinomas expressing the epithelial marker EpCAM and faster proliferating compact synaptophysin-positive neuroendocrine tumors in the colon of *Villin*-Cre-ER^T2^×LoxP-TAg mice after more than one year. Thus transdifferentiation appears to be a slow process possibly involving tedious remodeling of epigenetic modifications. Such is observed during reprogramming of adult cells to induced pluripotent stem cells (iPS) which can take several weeks [22]. On the other hand, most of the sparse TAg-positive tumor cells observed in 19- and 30-day-old *CEA424*-SV40 TAg mice already express neuroendocrine markers but no mucins as shown in 30-day-old transgenic mice (data not shown and Figure 1, Figure S1). We interpret these data as an indication that there is no direct transit from TAg-positive mucus-producing progenitor cells to cells expressing neuroendocrine markers beyond day 30 of development. In 19-day-old mice, single TAg-positive cells are found near the bottom of the gastric unit or close to the +4 position which is thought to harbor stem cells and gastric progenitor cells, respectively (E. V. and W. Z., unpublished results; [23]). These cells could more rapidly differentiate into cells committed to the neuroendocrine lineage alleviating the need for an extended period of time for the development of neuroendocrine tumors in this model. Despite the pronounced differences in kinetics and number of possible differentiation steps involved in the acquisition of the neuroendocrine phenotype, invasive tumors in both *CEA424*-SV40 TAg and *ATP4b*-SV40 TAg mice share a surprisingly similar neuroendocrine gene expression signature (Syder signature [4]) including a set of common transcription factors. Interestingly, in both models, invasive gastric carcinomas share expression of the transcriptional regulator gene *Etv1* which has recently been shown to encode a master regulator in neuronally derived GIST tumors and is commonly affected by gene translocations in prostate tumors which often exhibit a neuroendocrine phenotype upon progression [18], [24], [25].

But, why do not all SV40 TAg models develop neuroendocrine tumors? This might be due to expression of TAg in cells with a rapid turn-over which would not leave enough time for transdifferentiation. Indeed, in *Fabp2/I-FABP*-SV40 TAg mice mainly hyperplasia is observed [19]. In this model TAg enforces the reentry of intestinal enterocytes into the cell cycle and induces suppression of apoptosis by inactivation of RB and p53, respectively. Similarly, when *Apc*, a key gatekeeper of intestinal tumorigenesis, is deleted in short-lived transit-amplifying cells in colonic crypts using *Ah*-Cre×loxP-*Apc* mice, the growth of the induced microadenomas rapidly subsides while inactivation of *Apc* in long-lived colonic stem cells leads to persistent adenomas [26].

Preferential induction of the neuroendocrine phenotype is possibly not restricted to SV40 TAg. Most interestingly, epidemiological and molecular genetic studies demonstrated a high prevalence (80%) of a recently discovered human polyomavirus (Merkel cell polyomavirus, MCPyV) in Merkel cell carcinomas, a rare aggressive skin tumor with epithelial and neuroendocrine features [27], [28]. MCPyV, like the related SV40 virus, encodes a large T antigen which has been shown to bind to the RB protein and is needed for the maintenance of the growth capability of the Merkel carcinoma cells [29]. It is assumed that MCPyV TAg also inactivates the function of both p53 and RB as it is known for the large T antigen of SV40 TAg.

To analyze whether TAg might be more directly involved in establishment of the neuroendocrine phenotype we downregulated TAg expression by siRNA in TAg-expressing 424GC gastric cancer cells. As expected, classical E2F target genes (Cantalupo E2F gene signature [19]) were downregulated after TAg depletion and concomitant relief of RB repression. Notably, the expression of genes found to be typically active in neuroendocrine tumors (Hofsli gene signature [15]) were also significantly diminished in 424GC cells, including genes encoding neuroendocrine markers such as chromogranin A, dopa decarboxylase (DDC) and TPH1. The mRNA of the transcriptional regulator gene *Hey1* known to be under E2F control [20] was also significantly reduced by TAg siRNA and thus could possibly convey changes in expression of genes encoding neuroendocrine factors. However, mRNA levels of other transcription factor genes which regulate the neuroendocrine lineage, like *Nkx2.2* and *Neurod1*, were not significantly altered by TAg siRNA. Thus the mechanism of downregulation of neuroendocrine gene signature remains unclear.

The neuroendocrine phenotype observed recurrently in tumors of SV40 TAg-transgenic mice suggests that simultaneous inactivation of p53 and RB proteins, a hallmark of SV40 TAg action, favors the development of neuroendocrine tumors. Indeed, tumors with neuroendocrine features predominantly develop in genetically manipulated mouse strains where *Trp53* and *Rb* family genes have been inactivated. For example, after conditional inactivation of *Trp53* and *Rb* and optionally of the Rb-related gene *p130* in lung epithelia by application of Cre recombinase-expressing adenoviral vectors, mice succumbed to neuroendocrine lung tumors [30]. A set of 23 genes most strongly overexpressed in lung tumors of double- as well as in triple-mutant mice in comparison to normal lung tissue are also overexpressed both in human small cell lung carcinomas which also display a neuroendocrine phenotype and in *CEA*424-SV40 TAg gastric tumors as shown by GSEA (Table 1). Among others, those genes encode typical neuroendocrine markers like CHGA, CHGB, DDC and CALCA [30]. Most interestingly, this neuroendocrine gene signature is also significantly downregulated by TAg siRNA treatment in 424GC cells (Table 1). This suggests that p53 and RB play a decisive role in the establishment of the neuroendocrine phenotype in 424GC cells. Furthermore, specific conditional ablation of both *Rb* and *Trp53* in osteoblast precursors using an *osterix* promoter-Cre deleter strain induced in 75% of the double knock-out mice metastatic osteosarcomas but, most remarkable, in 60% of the mice metastatic neuroendocrine tumors were detected as well [31]. In addition, inactivation of both tumor suppressor genes in prostate epithelium based on a composite rat probasin promoter-driven Cre expression resulted in rapidly growing invasive and metastatic prostate carcinomas showing both luminal epithelial and neuroendocrine differentiation [32]. Interestingly, high frequency of simultaneous loss of *p53* and *RB1* alleles is regularly observed in human small cell lung carcinomas as well as in large cell neuroendocrine carcinomas of the lung, which are both tumors with neuroendocrine marker expression [33]. Thus induction of the neuroendocrine phenotype could also be linked to the loss of the tumor suppressors p53 and RB1 in human tumors. However, loss of *Trp53* and *Rb* gene function is not always associated with the neuroendocrine tumor phenotype. Targeted deletion of these two genes in stem/bipotent progenitors of breast epithelia using mouse mammary tumor virus LTR-driven Cre expression led to histologically uniform, aggressive breast tumors with an epithelial to mesenchymal transition (EMT) phenotype [34]. Furthermore, deletion of both tumor suppressor genes in mesenchymal limb bud cells induced sarcomas in mice [35], [36]. This might hint towards tissue specificity of the action of p53 and Rb and/or involvement of additional SV40 TAg target genes on top of *Trp53* and *Rb*, like the *p300* gene in the generation of neuroendocrine tumors [1].

### Conclusions

Taken together, strong evidence has accumulated that simultaneous inactivation of p53 and RB either by TAg or genetic alterations can transform cells of various cell lineages, often of epithelial origin, into tumor cells with neuroendocrine characteristics. Although less likely, preferential expression of SV40 TAg or selective sensitivity of neuroendocrine progenitor cells towards SV40 TAg action cannot be rigorously excluded. To clarify these issues, more defined conditional murine models are needed which allow reproducible targeting of stem cells, progenitor cells or differentiated cells in adult mice using e.g. loxP-SV40 TAg mice in combination with cell type-specific knock-in Cre-ER^T2^ deleter strains [26], [37].

## Methods

### Ethics statement

All animal work has been conducted according to relevant national and international guidelines. Animal studies within this work were registered with and accredited by the local regulatory agency (Regierung von Oberbayern, Munich, Germany) with registration number G91.

### Mice

The *CEA424*-SV40 TAg mice [12] were kept and bred at the animal facility of the Walter Brendel Centre of Experimental Medicine, Munich, Germany. They were maintained hemizygously on a C57BL/6 background.

### Size determination of tumors

Stomachs were opened along the small curvature, flushed with ice-cold PBS, pinned with needles to a cork disk, fixed in PBS-buffered 4% formaldehyde for 2 h at room temperature, dehydrated and soaked in paraffin wax. The stomachs were sectioned in 4 stripes of equal width along the anterior/posterior axis, which were paraffin wax embedded after clock-wise rotation for 90°. Tissue sections were periodic acid Schiff (PAS) stained to reveal the PAS negative tumors. Tumor area was determined using the AxioVision Digital Image Processing Software (Carl Zeiss MicroImaging GmbH, Jena, Germany) and displayed as mean ± SD.

### Tumor Cell Lines

The cell lines 424GC, mGC3 and mGC8 that were used in this study have been generated earlier from tumors of *CEA424*-SV40 TAg mice [13]. The cells were grown in RPMI1640 with sodium pyruvate, non-essential amino acids, glutamine, 50 µM 2-mercaptoethanol, 10% fetal bovine serum (Lonza, Cologne, Germany). The murine endothelioma cell line bEnd.3 was obtained from D. Vestweber, MPI Münster) and cultured as above.

### Western Blot

Cells and tumor tissue were lysed in RIPA buffer with protease inhibitors (Complete Mini, Roche Penzberg, Germany). Protein concentrations were measured using the Bradford assay (Bio-Rad Laboratories GmbH, Munich, Germany). Equal protein amounts (100 µg per sample) were separated on 8–16% sodium dodecyl sulfate polyacrylamide gels (Thermo-Fisher, Bonn, Germany) by electrophoresis followed by transfer to nitrocellulose. Membranes were probed with either a monoclonal rabbit anti-NEUROD1 antibody (# 4373, New England Biolabs/Cell Signaling, Frankfurt, Germany), polyclonal rabbit antibodies against SV40 TAg (sc-20800; Santa Cruz, Heidelberg, Germany) or a rabbit anti-β-actin (sc-1616, Santa Cruz) followed by incubation with a horse radish peroxidase (HRP)-tagged anti-rabbit IgG antibody (New England Biolabs/Cell Signaling). For HRP detection SuperSignal West Pico Chemiluminescent Substrate (Thermo-Fisher) was used according to the supplier's protocol.

### ELISA

Serotonin concentrations were measured in EDTA plasma by a competition ELISA (IBL, Hamburg, Germany) according to the manufacture's protocol.

### Immunohistochemistry and immunofluorescence

Stomachs were fixed and embedded as above. Serial sections were dewaxed and stained with the appropriate antibodies after heat retrieval of the antigens at pH 9.0 (Target Retrieval Solution; Dako Deutschland GmbH, Hamburg, Germany). The tissue sections were reacted with primary antibodies that were visualized using HRP-coupled secondary antibodies and 3-amino-9-ethylcarbazole as substrate (ImmPRESS Anti-Rabbit Ig Polymer Detection Kit; Vector Labs, Burlington, Ontario, Canada). Sections were counterstained with hematoxylin and viewed with a Nikon Eclipse E800 microscope and photographed using a Nikon DS-5M-L1 digital camera. For immunofluorescence, tissue sections were blocked for 20 min in 2.5% horse serum. Incubation with primary antibody was at 4°C overnight or at room temperature for 2 h. After washing with PBS, secondary antibodies, conjugated to Alexa-568 or Alexa-647 (Invitrogen) were added for 1 h at room temperature. Sections were counterstained with DAPI (0.5 µg/ml) and mounted in Dako Mounting Medium (Dako). Imaging was performed with a Leica DM IRBE scanning confocal microscope or a Zeiss Axiophot microscope. The following primary antibodies were used: polyclonal goat antibodies against chromogranin A (1∶200; sc-1488; Santa Cruz), polyclonal rabbit antibodies against SV40 TAg (1∶500; sc-20800; Santa Cruz). After SV40 TAg detection by immunohistochemistry mucin-secreting cells were stained by incubation with 1% Alcian blue/3% acetic acid solution pH 2.5 for 30 min.

### Electron microscopy

Tumor samples (0.5 to 1 mm^3^ pieces) were immersed in 3.5% glutardialdehyde. Post-fixation treatment was performed in 1% OsO_4_ containing 1.5% K_4_Fe(CN)_6_ in 0.1 M cacodylate buffer for 1 h. After dehydration and embedding in Araldite (SERVA Electrophoresis GmbH, Heidelberg, Germany) sections were cut on a Leica Ultracut UCT ultramicrotome and examined in a Zeiss EM 900 transmission electron microscope.

### RNA isolation and reverse transcription PCR

Total RNA was isolated from tissue and cell samples using the NucleoSpin RNA II kit (Macherey-Nagel, Düren, Germany) according to the manufacturer's protocol. Standard protocols were applied for reverse transcription of RNA and detection of SV40 TAg cDNA by PCR. The following forward and reverse primers which flank the intron present in the SV40 TAg gene were used: 5′-AATTCTGAAGGAAAGTCCTTGG, 5′- TAATGGACCTTCTAGGTCTTGA.

### Microarray analysis

To characterize the gene expression profile in the stomach three mice each at an age of 30, 60 or 90 days were sacrificed. Tissues were taken from the antrum of the stomach where tumor lesions were visible macroscopically by the age of 60 days. Tissues from the same anatomical region of three non-transgenic mice were used as control. Total RNA was isolated as described above. Biotinylated cRNA was prepared with the Illumina® TotalPrep™ RNA Amplification Kit (Applied Biosystems, Darmstadt, Germany). cRNA (1.5 µg) was hybridized to Sentrix Mouse-6 Expression Beadchips (Illumina, San Diego, CA) and scanned on Illumina BeadStation 500×. For data collection Illumina BeadStudio software was used. The data were normalized using R Statistical language and packages from the Bioconductor project as well as the vsn normalization method [38]. Microarray data comply with MIAME guidelines and are available at http://www.ncbi.nlm.nih.gov/geo (accession no. GSE27712). Microarray data were analyzed for enrichment of preselected gene sets by Gene Set Enrichment Analysis (GSEA), developed by the Broad-Institute, Cambridge, MA 02141, USA (http://broad.harvard.edu/gsea/) [39], [40]. Differentially expressed genes were assessed by applying t-test with p<0.05, fold change (fc) filters (fc ≥2) and difference of means >100 using dChip software. For this purpose microarray raw data of the respective samples were normalized using quantiles normalization. Hierarchical clustering and principal component analysis (PCA) was performed based on genes differentially expressed between controls and 90-day-old transgenic mice (t-test with p<0.05, fc ≥2, difference of means >100 and passing 10% false discovery rate).

### siRNA experiments

Exponentially growing cells were transfected with siRNA using Lipofectamine™ (Invitrogen GmbH; Darmstadt, Germany). SV40 TAg-specific siRNA was designed using the BLOCK-iT™ RNAi Designer (Invitrogen). Three Stealth RNAi siRNA sequences were tested at different concentration and finally used at 600 nM in the experiments. After 24, 48 and 72 h, cells were lysed for protein analysis as described. For gene expression analysis RNA was isolated after 72 h as described above. RNA isolation and microarray analysis was performed as described above. Data were normalized using the quantile normalization method. Microarray data are available at http://www.ncbi.nlm.nih.gov/geo (accession no. GSE27712).

### Statistical analysis

Experimental data were analyzed by Sigma plot for Windows, Version 10.0 (Systat Software Inc., San Jose, USA) using Student's t-test. P-values of p<0.05 were considered as statistically significant.

## Supporting Information

Figure S1 SV40 TAg-positive tumor cells do not express mucins.
(PDF)

Table S1 Genes upregulated in the antrum of 90-day-old *CEA424*-SV40 TAg mice in comparison to the antrum of non-transgenic littermates.
(PDF)

Table S2 Fraction of neuroendocrine (NE) genes/probe sets up- or down-regulated in tumors of *CEA424*-SV40 TAg mice.
(PDF)

Table S3 Expression of transcription factor and DNA-binding protein genes from the Syder signature in d90 *CEA424*-SV40 TAg tumors.
(PDF)

Table S4 Genes deregulated in 424GC cells by SV40 TAg-specific siRNA.
(PDF)

Table S5 Neuroendocrine phenotype in SV40-TAg-transgenic mouse tumor models.
(PDF)

References S1 References for Table S5.
(PDF)
