# Supplementary material for: Expression of a Neuroendocrine Gene Signature in Gastric Tumor Cells from CEA 424-SV40 Large T Antigen-Transgenic Mice Depends on SV40 Large T Antigen
Source: PLoS One. 2012 Jan 13;7(1):e29846. doi: 10.1371/journal.pone.0029846 (PMC3258231; doi:10.1371/journal.pone.0029846)
Supplement: Figure S1 — SV40 TAg-positive tumor cells do not express mucins. (PDF) [file pone.0029846.s001.pdf]

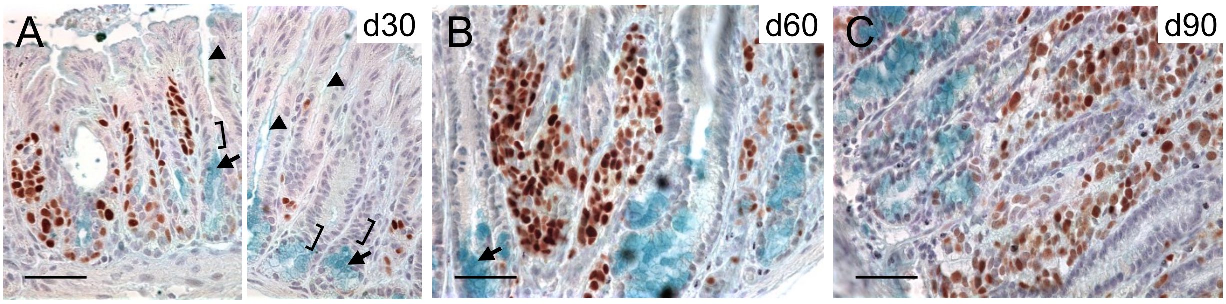

Figure S1: SV40 TAg-positive tumor cells do not express mucins. Paraffin sections of stomachs from 30 (A), 60 (B) and 90-day-old CEA424 SV40 TAg mice (C) were double-stained with Alcian blue for mucins and tumor cells were identified with anti-SV40 TAg antibodies (brown color). Note strong cytoplasmic Alcian blue staining in crypt cells below (arrows) and apical staining (arrowheads) above the isthmus region (marked by brackets) of the gastric glands, a staining pattern which is characteristic for mucin 6 and mucin 5ac, respectively (A). No Alcian blue staining was observed in SV40 TAg-positve cells. Magnification bars: 50 µm.
