# Supplementary material for: Expression of a Neuroendocrine Gene Signature in Gastric Tumor Cells from CEA 424-SV40 Large T Antigen-Transgenic Mice Depends on SV40 Large T Antigen
Source: PLoS One. 2012 Jan 13;7(1):e29846. doi: 10.1371/journal.pone.0029846 (PMC3258231; doi:10.1371/journal.pone.0029846)
Supplement: Table S1 — Genes upregulated in the antrum of 90-day-old CEA424-SV40 TAg mice in comparison to the antrum of non-transgenic littermates. (PDF) [file pone.0029846.s002.pdf]

**Table S1: Genes upregulated in the antrum of 90-day-old *CEA424* -SV40 TAg mice in comparison to the antrum of non-transgenic littermates**

| Gene[1] | Description | Probe set | Fluorescence d90 tumor | Fold change | P value[2] |
|---|---|---|---|---|---|
| **Chgb** | chromogranin B (Chgb), mRNA. | scl20253.5_8-S | 25374 | 192.3 | **0.012** |
| **Tph1** | tryptophan hydroxylase 1 (Tph1), mRNA. | scl31346.12.1_18-S | 5582 | 146.1 | **0.010** |
| **Calca** | calcitonin/calcitonin-related polypeptide, alpha (Calca), mRNA. | scl0012310.2_237-S | 2082 | 113.9 | 0.063 |
| **Gcg** | glucagon (Gcg), mRNA. | scl19223.5.1_16-S | 6607 | 110.9 | **0.007** |
| **Scg2** | secretogranin II (Scg2), mRNA. | scl0020254.2_71-S | 8695 | 110.6 | **0.014** |
| Dlk1 | delta-like 1 homolog (Drosophila) (Dlk1), mRNA. | scl42790.6_48-S | 5917 | 87.8 | **0.000** |
| Sbp | spermine binding protein (Sbp), mRNA. | scl50224.3.1_12-S | 1747 | 74.9 | 0.052 |
| **Sct** | secretin (Sct), mRNA. | scl30493.4.19_120-S | 26665 | 68.9 | **0.040** |
| **Grp** | gastrin releasing peptide (Grp), mRNA. | scl51842.4.1_165-S | 1785 | 49.7 | **0.013** |
| **Pappa2** | 9430076M13 | scl00329275.1_98-S | 2330 | 47.2 | **0.038** |
| **Calca** | calcitonin/calcitonin-related polypeptide, alpha (Calca), mRNA. | scl30784.6.1_78-S | 1327 | 46.3 | 0.091 |
| 1810009J06Rik | RIKEN cDNA 1810009J06 gene (1810009J06Rik), mRNA. | scl30151.5.1_76-S | 6914 | 44.6 | 0.255 |
| **Gad2** | glutamic acid decarboxylase 2 (6330404F12Rik), mRNA | scl21216.1.1627_35-S | 1352 | 42.8 | **0.022** |
| Hap1 | huntingtin-associated protein 1 (Hap1), transcript variant 1, mRNA. | scl39565.12_79-S | 4465 | 40.2 | **0.015** |
| **Syt4** | synaptotagmin 4 (Syt4), mRNA. | scl0020983.2_127-S | 1457 | 38.6 | **0.010** |
| Nt5dc2 | 5'-nucleotidase domain containing 2 (2510015F01Rik), mRNA | ri\|2510015F01\|ZX00047N 14\|AK010966\|669-S | 4741 | 37.0 | **0.018** |
| Igh-1a | | scl0380793.1_210-S | 2115 | 36.7 | **0.048** |
| Dlk1 | delta-like 1 homolog (Drosophila) (Dlk1), mRNA. | scl0013386.2_11-S | 2164 | 35.9 | **0.006** |
| Miat | myocardial infarction associated transcript (non-protein coding) (A230057G18Rik) | scl26213.1.3_304-S | 1058 | 35.6 | 0.073 |
| 2310051P22Rik | | ri\|2310051P22\|ZX00059F 14\|AK075905\|599-S | 2416 | 32.6 | 0.080 |
| **Snap25** | synaptosomal-associated protein 25 (Snap25), mRNA. | scl20231.9_372-S | 4661 | 29.3 | **0.027** |
| **Sgne1/Scg5** | secretory granule neuroendocrine protein 1, 7B2 protein (Sgne1), mRNA. | scl18858.8.1_25-S | 3446 | 29.3 | **0.033** |
| **Pdyn** | prodynorphin (Pdyn), mRNA. | scl18671.5_48-S | 2589 | 29.0 | **0.004** |
| Amigo2 | adhesion molecule with Ig like domain 2 (AI415330), mRNA. | scl46785.2.1_0-S | 1969 | 28.2 | **0.044** |
| Kndc1 | kinase non-catalytic C-lobe domain (KIND) containing 1 (2410012C07Rik), mRNA. | scl076484.1_294-S | 941 | 27.8 | **0.038** |
| **Resp18** | regulated endocrine-specific protein 18 (Resp18), mRNA. | scl16596.6.1_82-S | 12005 | 27.5 | **0.017** |
| Thy1 | thymus cell antigen 1, theta (Thy1), mRNA. | scl37046.4_393-S | 3803 | 27.2 | **0.000** |
| Nptx2 | neuronal pentraxin 2 (Nptx2), mRNA. | scl26994.6.1_84-S | 938 | 27.1 | **0.001** |
| Amigo2 | adhesion molecule with Ig like domain 2 (AI415330), mRNA. | scl00105827.2_285-S | 3032 | 26.5 | **0.045** |
| Nt5dc2 | 5'-nucleotidase domain containing 2 (2510015F01Rik), mRNA | scl46497.11.10_2-S | 940 | 25.7 | **0.003** |
| Rufy3 | RUN and FYVE domain containing 3 (D5Bwg0860e), mRNA. | scl0052822.2_239-S | 758 | 25.5 | **0.026** |
| Wif1 | Wnt inhibitory factor 1 (Wif1), mRNA. | scl38368.11_94-S | 970 | 25.1 | **0.015** |
| B930041J05 | 10 days neonate cerebellum cDNA, RIKEN full-length enriched library, clone:B930041J05 product:unclassifiable, full insert sequence. | scl0003318.1_26-S | 953 | 24.1 | **0.029** |

| | | | | | |
|---|---|---|---|---|---|
| Igh-4 | | scl0016017.1_1-S | 497 | 23.2 | 0.076 |
| Stk32a | serine/threonine kinase 32A | scl52018.13_498-S | 943 | 22.4 | **0.030** |
| Cck | cholecystokinin (Cck), mRNA. | scl35202.5.1_0-S | 823 | 21.2 | **0.024** |
| Nkx2-2 | NK2 transcription factor related, locus 2 (Drosophila) (Nkx2-2), mRNA. | scl0018088.2_84-S | 490 | 21.1 | **0.010** |
| Trrp7 | transient receptor protein 7 | ri\|9830168K16\|PX00119C18\|AK036731\|3991-S | 573 | 21.0 | **0.040** |
| Mdm1 | transformed mouse 3T3 cell double minute 1 (Mdm1), mRNA. | scl38394.14.1_0-S | 957 | 20.9 | 0.068 |
| Cryba2 | crystallin, beta A2 (Cryba2), mRNA. | scl16609.5.1_17-S | 1272 | 20.5 | **0.017** |
| Insm1 | insulinoma-associated 1 (Insm1), mRNA. | scl20175.1.1_43-S | 1037 | 20.4 | **0.000** |
| Dner | delta/notch-like EGF-related receptor (Dner), mRNA. | scl16535.14_269-S | 1491 | 20.1 | **0.025** |
| Sphkap | SPHK1 interactor, AKAP domain containing (4930544G21Rik), mRNA. | scl16544.13_20-S | 824 | 19.6 | **0.028** |
| Eef1a2 | eukaryotic translation elongation factor 1 alpha 2 (Eef1a2), mRNA. | scl013628.1_321-S | 2752 | 19.6 | **0.004** |
| Tmem27 | transmembrane protein 27 | scl54490.7_1-S | 603 | 19.6 | **0.049** |
| Ighg2c | immunoglobulin heavy constant gamma 2C | IGHG2C_J00479_Ig_heavy_constant_gamma_2C_715-S | 669 | 19.0 | 0.058 |
| Mdm1 | transformed mouse 3T3 cell double minute 1 (Mdm1), mRNA. | scl0003883.1_511-S | 700 | 18.9 | 0.074 |
| Siat8e | sialyltransferase 8 (alpha-2, 8-sialyltransferase) E (Siat8e), transcript variant 2, mRNA. | scl51743.8.1_178-S | 436 | 18.9 | **0.007** |
| Pcsk2 | proprotein convertase subtilisin/kexin type 2 (Pcsk2), mRNA. | scl018549.12_191-S | 1547 | 18.8 | **0.013** |
| Plk5 | polo-like kinase 5 (Drosophila) (6330514A18Rik), mRNA. | scl38681.14.1_267-S | 831 | 18.3 | **0.041** |
| Nov | nephroblastoma overexpressed gene (Nov), mRNA. | scl47911.5_100-S | 538 | 18.2 | **0.021** |
| Serpina3n | serine (or cysteine) proteinase inhibitor, clade A, member 3N (Serpina3n), mRNA. | scl020716.5_261-S | 4118 | 17.9 | **0.039** |
| Wisp2 | WNT1 inducible signaling pathway protein 2 (Wisp2), mRNA. | scl19952.5.1_24-S | 504 | 17.8 | **0.001** |
| B3gat1 | beta-1,3-glucuronyltransferase 1 (glucuronosyltransferase P) (B3gat1), mRNA. | scl0076898.1_65-S | 435 | 17.3 | **0.011** |
| Etv1 | ets variant gene 1 (Etv1), mRNA. | scl014009.13_0-S | 2437 | 17.3 | **0.014** |
| Spc25 | SPC25, NDC80 kinetochore complex component, homolog (S. cerevisiae) (2600017H08Rik), mRNA. | scl19174.6.1_6-S | 2097 | 17.2 | **0.016** |
| Celsr3 | cadherin EGF LAG seven-pass G-type receptor 3 (Celsr3), mRNA. | scl0107934.22_249-S | 561 | 16.9 | **0.013** |
| Gprasp2 | G protein-coupled receptor associated sorting protein 2 | scl54621.6_157-S | 1260 | 16.6 | **0.032** |
| Asphd1 | aspartate beta-hydroxylase domain containing 1 (A830007L07Rik), mRNA | scl30664.4.1_54-S | 338 | 16.5 | **0.010** |
| Brunol4 | bruno-like 4, RNA binding protein (Drosophila) (Brunol4), mRNA. | scl51577.15_266-S | 2781 | 16.4 | **0.026** |
| Dscam | Down syndrome cell adhesion molecule (Dscam), mRNA. | scl0013508.1_59-S | 321 | 16.3 | **0.030** |
| Nxph1 | neurexophilin 1 (Nxph1), mRNA. | scl30391.3_48-S | 681 | 16.3 | **0.033** |
| Bex2 | brain expressed X-linked 2 (Bex2), mRNA. | scl53819.3_141-S | 5535 | 16.0 | **0.005** |
| **Sv2a** | synaptic vesicle glycoprotein 2 a (Sv2a), mRNA. | scl064051.13_189-S | 712 | 15.9 | **0.015** |
| Lrp11 | low density lipoprotein receptor-related protein 11 (Lrp11), mRNA. | scl39172.7_550-S | 1349 | 15.9 | **0.014** |
| Pak3 | p21 (CDKN1A)-activated kinase 3 (Pak3), mRNA. | scl018481.14_0-S | 1068 | 15.9 | **0.013** |
| Zcchc18 | zinc finger, CCHC domain containing 18 (1500031H04Rik), mRNA. | scl54605.3_321-S | 1448 | 15.9 | **0.018** |
| **Gria2** | glutamate receptor, ionotropic, AMPA2 (alpha 2) (Gria2), mRNA. | scl0014800.2_192-S | 842 | 15.7 | **0.028** |
| **Chga** | chromogranin A (Chga), mRNA. | scl42855.7.114_47-S | 5147 | 15.6 | **0.013** |

| | | | | | |
|---|---|---|---|---|---|
| Rit2 | Ras-like without CAAX 2 (Rit2), mRNA. | scl0019762.2_135-S | 1075 | 14.9 | **0.016** |
| AI427515 | expressed sequence AI427515 (AI427515), mRNA. | scl33299.10_656-S | 1237 | 14.5 | **0.012** |
| Snhg11 | small nucleolar RNA host gene 11 (E130013N09Rik), mRNA | scl20000.1_138-S | 824 | 14.5 | **0.025** |
| Ighg1 | immunoglobulin heavy constant gamma 1 | IGHG1_J00453$V00793_Ig_heavy_constant_gamma_1_792 | 346 | 14.5 | 0.068 |
| Entpd3 | ectonucleoside triphosphate diphosphohydrolase 3 (Entpd3), mRNA. | scl36335.20.967_4-S | 2446 | 14.5 | 0.070 |
| C130036G08 | hypothetical protein C130036G08 (C130036G08), mRNA. | scl25790.8_644-S | 653 | 14.3 | **0.018** |
| Slc35d3 | solute carrier family 35, member D3 | scl38150.2.1_277-S | 530 | 14.1 | **0.012** |
| **Ddc** | dopa decarboxylase (Ddc), mRNA. | scl40512.21.1_20-S | 3022 | 14.1 | **0.002** |
| Gap43 | growth associated protein 43 (Gap43), mRNA. | scl014432.1_11-S | 854 | 13.9 | **0.015** |
| C630025L14 | hypothetical protein C630025L14 (C630025L14), mRNA. | scl00210980.1_280-S | 547 | 13.9 | **0.013** |
| Mfng | manic fringe homolog (Drosophila) (Mfng), mRNA. | scl46983.8_85-S | 914 | 13.8 | **0.010** |
| **Syt1** | synaptotagmin 1 (Syt1), mRNA. | scl37517.13_240-S | 762 | 13.8 | **0.030** |
| Trpc4 | transient receptor potential cation channel, subfamily C, member 4 (Trpc4), mRNA. | scl23196.13.253_8-S | 489 | 13.4 | **0.018** |
| Pacsin1 | protein kinase C and casein kinase substrate in neurons 1 (Pacsin1), mRNA. | scl0023969.1_255-S | 530 | 13.3 | **0.015** |
| Saa3 | serum amyloid A 3 (Saa3), mRNA. | scl31343.5.1_35-S | 944 | 13.3 | 0.123 |
| A2bp1 | ataxin 2 binding protein 1 (A2bp1), transcript variant 2, mRNA. | scl0268859.12_21-S | 482 | 13.2 | **0.025** |
| Stxbp1 | syntaxin binding protein 1 (Stxbp1), mRNA. | scl020910.1_1-S | 3019 | 13.1 | **0.015** |
| Sez6l | seizure related 6 homolog like | scl26208.17_33-S | 410 | 13.0 | 0.079 |
| Tro | trophinin (Tro), mRNA. | scl53734.13.1_1-S | 879 | 13.0 | **0.032** |
| Gm691 | gene model 691, (NCBI) (Gm691), mRNA. | scl0277432.5_328-S | 1095 | 12.9 | **0.017** |
| **Ddc** | dopa decarboxylase (Ddc), mRNA. | scl0013195.2_156-S | 2551 | 12.7 | **0.004** |
| 2700016F22Rik | RIKEN cDNA 2700016F22 gene | scl2206.1.1_322-S | 619 | 12.6 | 0.112 |
| **Gabrb3** | gamma-aminobutyric acid (GABA-A) receptor, subunit beta 3 (Gabrb3), mRNA. | scl32600.10_229-S | 898 | 12.5 | **0.027** |
| Chst1 | carbohydrate (keratan sulfate Gal-6) sulfotransferase 1 (Chst1), mRNA. | scl076969.2_187-S | 1782 | 12.5 | **0.010** |
| Ighg | Immunoglobulin heavy chain (gamma polypeptide) (AU044919) | scl0380794.2_30-S | 451 | 12.3 | 0.100 |
| Ptprn | protein tyrosine phosphatase, receptor type, N (Ptprn), mRNA. | scl16598.23.1_93-S | 531 | 12.2 | **0.007** |
| 3110018K01 | 13 days embryo head cDNA, RIKEN full-length enriched library, clone:3110018K01 product:unknown EST, full insert sequence. | scl0002540.1_6-S | 465 | 12.2 | **0.012** |
| Prss12 | protease, serine, 12 neurotrypsin (motopsin) (Prss12), mRNA. | scl22657.12.1_29-S | 548 | 12.1 | **0.018** |
| Rgs9 | regulator of G-protein signaling 9 (Rgs9), mRNA. | scl39397.22.1_286-S | 886 | 11.9 | **0.022** |
| Peg3 | paternally expressed 3 (Peg3), mRNA. | scl018616.1_273-S | 851 | 11.9 | 0.077 |
| **Chga** | chromogranin A (Chga), mRNA. | scl0002400.1_38-S | 3278 | 11.8 | **0.025** |
| Tagln3 | transgelin 3 (Tagln3), mRNA. | scl48442.3.1_5-S | 518 | 11.8 | **0.010** |
| **Eno2** | enolase 2, gamma neuronal (Eno2), mRNA. | scl013807.1_30-S | 1170 | 11.5 | **0.019** |
| Csen | calsenilin, presenilin binding protein, EF hand transcription factor (Csen), mRNA. | scl18706.10.1_133-S | 366 | 11.5 | **0.009** |
| Hp | haptoglobin (Hp), mRNA. | scl34345.7_19-S | 1096 | 11.5 | 0.077 |
| 2700045K19Rik | | ri\|2700045K19\|ZX00056N06\|AK012378\|949-S | 456 | 11.4 | **0.024** |
| Fam183b | family with sequence similarity 183, member B (Fam183b), mRNA | scl40179.4.1_10-S | 939 | 11.4 | 0.054 |

| | | | | | |
|---|---|---|---|---|---|
| Pcsk2 | proprotein convertase subtilisin/kexin type 2 (Pcsk2), mRNA. | scl20208.12.1_29-S | 621 | 11.4 | **0.004** |
| Trim9 | tripartite motif-containing 9 (Trim9), mRNA | scl0002358.1_1625-S | 248 | 11.3 | **0.026** |
| **Gria2** | glutamate receptor, ionotropic, AMPA2 (alpha 2) (Gria2), mRNA. | scl22034.13_42-S | 413 | 11.3 | 0.089 |
| AW121567 | expressed sequence AW121567 (AW121567), mRNA. | scl35118.3_474-S | 623 | 11.3 | **0.034** |
| Crtac1 | cartilage acidic protein 1 (Crtac1), mRNA. | scl52473.16_34-S | 314 | 11.2 | **0.000** |
| E430021N18Rik | RIKEN cDNA E430021N18 gene (E430021N18Rik), mRNA. | scl17113.9_435-S | 405 | 11.1 | **0.037** |
| **Syp** | synaptophysin (Syp), mRNA. | scl020977.7_194-S | 2030 | 11.1 | **0.013** |
| Stmn3 | stathmin-like 3 (Stmn3), mRNA. | scl020262.2_8-S | 589 | 10.9 | **0.014** |
| AV344025 | expressed sequence AV344025 (AV344025), mRNA. | scl49565.1.1_305-S | 367 | 10.9 | **0.020** |
| C1qtnf4 | C1q and tumor necrosis factor related protein 4 (C1qtnf4), mRNA. | scl20653.2_227-S | 1024 | 10.8 | **0.015** |
| Elavl3 | ELAV (embryonic lethal, abnormal vision, Drosophila)-like 3 (Hu antigen C) | scl066572.1_0-S | 424 | 10.8 | **0.013** |
| 2900063K03Rik | RIKEN cDNA 2900063K03 gene | scl26306.1.1_82-S | 496 | 10.6 | 0.144 |
| Pcsk1n | proprotein convertase subtilisin/kexin type 1 inhibitor (Pcsk1n), mRNA. | scl0030052.2_159-S | 930 | 10.6 | **0.016** |
| St18 | suppression of tumorigenicity 18 (St18), mRNA. | scl18181.27.1_309-S | 470 | 10.4 | **0.014** |
| Rgag4 | retrotransposon gag domain containing 4 | scl53982.1_257-S | 363 | 10.4 | **0.022** |
| Thbs2 | thrombospondin 2 (Thbs2), mRNA. | scl0021826.1_238-S | 913 | 10.4 | **0.023** |
| Trim35 | tripartite motif-containing 35 (Trim35), mRNA. | scl46157.6_151-S | 339 | 10.3 | **0.046** |
| Ddx25 | DEAD (Asp-Glu-Ala-Asp) box polypeptide 25 (Ddx25), mRNA. | scl36044.11.1_77-S | 398 | 10.1 | **0.029** |
| A930011O12Rik | RIKEN cDNA A930011O12 gene | scl46184.5_151-S | 240 | 10.1 | 0.069 |
| 6430402H13Rik | RIKEN cDNA 6430402H13 gene (6430402H13Rik), mRNA. | scl0378937.1_229-S | 396 | 10.1 | **0.007** |
| Camk2n2 | calcium/calmodulin-dependent protein kinase II inhibitor 2 | scl48661.2.1_28-S | 2245 | 10.0 | **0.029** |
| Tro | trophinin (Tro), mRNA. | scl0056191.2_129-S | 503 | 10.0 | **0.022** |
| Galr1 | galanin receptor 1 (Galr1), mRNA. | scl51205.4.8_60-S | 197 | 9.9 | **0.041** |
| Sult4a1 | sulfotransferase family 4A, member 1 (Sult4a1), mRNA. | scl46888.9_276-S | 780 | 9.8 | **0.007** |
| Gprin1 | G protein-regulated inducer of neurite outgrowth 1 (Gprin1), mRNA. | scl026913.1_318-S | 461 | 9.7 | **0.003** |
| Spock1 | sparc/osteonectin, cwcv and kazal-like domains proteoglycan 1 (Spock1), mRNA. | scl0020745.1_153-S | 406 | 9.7 | **0.012** |
| Spock2 | sparc/osteonectin, cwcv and kazal-like domains proteoglycan 2 | scl0014480.1_16-S | 199 | 9.7 | **0.028** |
| Inpp5f | inositol polyphosphate-5-phosphatase F (Inpp5f), mRNA. | scl31997.22_366-S | 2277 | 9.5 | **0.027** |
| Olfml3 | olfactomedin-like 3 | scl21737.4_335-S | 1734 | 9.5 | **0.016** |
| **Syn1** | synapsin I (Syn1), mRNA. | scl0020964.1_0-S | 416 | 9.5 | **0.012** |
| **Nsg2** | neuron specific gene family member 2 (Nsg2), mRNA. | scl41730.11_1-S | 356 | 9.5 | **0.006** |
| 4931429I11Rik | RIKEN cDNA 4931429I11 gene (4931429I11Rik), mRNA. | scl0003510.1_178-S | 261 | 9.4 | **0.030** |
| **Calcr** | calcitonin receptor (Calcr), mRNA. | scl29325.17.1_48-S | 279 | 9.4 | **0.009** |
| Ndrg4 | N-myc downstream regulated gene 4 (Ndrg4), mRNA | scl0234593.14_96-S | 1092 | 9.4 | **0.028** |
| Cntnap4 | contactin associated protein 4 (Cntnap4), mRNA. | scl33308.26.1_5-S | 503 | 9.2 | **0.020** |
| Iqsec3 | IQ motif and Sec7 domain 3 (Iqces3), mRNA | scl28446.23_488-S | 312 | 9.2 | **0.029** |
| Cplx1 | complexin 1 (Cplx1), mRNA. | scl26249.4_662-S | 535 | 9.1 | **0.007** |
| Olfm1 | olfactomedin 1 (Olfm1), mRNA. | scl0003187.1_40-S | 1567 | 9.1 | **0.012** |
| Dusp4 | dual specificity phosphatase 4 (Dusp4), mRNA. | scl279.1.1_54-S | 968 | 9.1 | **0.009** |
| S100a8 | S100 calcium binding protein A8 (calgranulin A) (S100a8), mRNA. | scl22940.2.1_25-S | 579 | 9.0 | 0.221 |
| Mdm1 | transformed mouse 3T3 cell double minute 1 (Mdm1), mRNA. | scl0003918.1_84-S | 163 | 8.9 | **0.042** |

| | | | | | |
|---|---|---|---|---|---|
| Otud7a | OTU domain containing 7A (Otud7a), mRNA | scl32590.14.1_290-S | 259 | 8.9 | **0.027** |
| Kcnc4 | potassium voltage gated channel, Shaw-related subfamily, member 4 (Kcnc4) | scl21680.4_681-S | 441 | 8.9 | **0.022** |
| Tmem59l | transmembrane protein 59-like (Tmem59l), mRNA | scl34700.7.1_46-S | 274 | 8.9 | **0.006** |
| LOC381283 | hypothetical gene supported by AK083095 (LOC381283), mRNA. | GI_38050350-S | 1894 | 8.9 | **0.026** |
| 4930488B01Rik | | scl33036.2.115_8-S | 335 | 8.9 | **0.022** |
| B130017P16Rik | RIKEN cDNA B130017P16 gene (B130017P16Rik), mRNA. | scl35423.7.1_70-S | 410 | 8.9 | **0.018** |
| Cadps | Ca<2+>dependent activator protein for secretion (Cadps), mRNA. | scl027062.1_219-S | 339 | 8.9 | **0.013** |
| Astn1 | astrotactin 1 (Astn1), mRNA. | scl000795.1_2960-S | 416 | 8.7 | **0.005** |
| C230081G24Rik | | ri\|C230081G24\|PX00176 N17\|AK048916\|1990-S | 219 | 8.6 | 0.082 |
| 1700086L19Rik | RIKEN cDNA 1700086L19 gene (1700086L19Rik), mRNA. | scl43092.8.1_221-S | 484 | 8.6 | **0.010** |
| Ttbk1 | tau tubulin kinase 1 (Ttbk1), mRNA | scl49868.1.1_330-S | 335 | 8.5 | **0.006** |
| Egr4 | early growth response 4 (Egr4), mRNA. | scl860.2.1_228-S | 200 | 8.5 | **0.000** |
| Gprasp1 | G protein-coupled receptor associated sorting protein 1 (Gprasp1), mRNA | scl54622.12_49-S | 1815 | 8.4 | **0.031** |
| Dnajc6 | DnaJ (Hsp40) homolog, subfamily C, member 6 (Dnajc6), mRNA. | scl0072685.1_143-S | 755 | 8.4 | **0.015** |
| Myt1l | myelin transcription factor 1-like (Myt1l), mRNA | scl3889.1.1_37-S | 309 | 8.3 | **0.048** |
| Cdkn2a | cyclin-dependent kinase inhibitor 2A (Cdkn2a), mRNA. | scl24121.4.1_71-S | 338 | 8.2 | **0.010** |
| Kcnb2 | potassium voltage gated channel, Shab-related subfamily, member 2 | GI_38049341-S | 352 | 8.2 | **0.035** |
| Pcsk2 | proprotein convertase subtilisin/kexin type 2 (Pcsk2), mRNA. | scl0018549.1_176-S | 664 | 8.2 | **0.011** |
| Arxes1 | adipocyte-related X-chromosome expressed sequence 1 (Arxes1), mRNA | scl54618.1.2_1-S | 878 | 8.2 | **0.003** |
| Rtn1 | reticulon 1 (Rtn1), mRNA. | scl42354.13_11-S | 420 | 8.1 | **0.043** |
| Hdc | histidine decarboxylase (Hdc), mRNA. | scl18719.12.1_11-S | 673 | 8.1 | **0.012** |
| Gja9 | gap junction membrane channel protein alpha 9 (Gja9), mRNA. | scl0014617.1_130-S | 177 | 8.1 | **0.024** |
| Diras1 | DIRAS family, GTP-binding RAS-like 1 (Diras1), mRNA. | scl0208666.1_329-S | 364 | 8.1 | **0.008** |
| Ddx26 | DEAD/H (Asp-Glu-Ala-Asp/His) box polypeptide 26 | ri\|D130058C17\|PX00185 E04\|AK051573\|2962-S | 1194 | 8.1 | **0.042** |
| Unc79 | unc-79 homolog (C. elegans) | scl42844.21.1_76-S | 333 | 8.1 | **0.031** |
| Atp6v0e2 | ATPase, H+ transporting, lysosomal V0 subunit E2 (VAtp6v0e2), mRNA | scl30088.4_382-S | 950 | 8.1 | **0.024** |
| Apof | apolipoprotein F (Apof), mRNA. | scl38289.2_239-S | 385 | 8.1 | 0.072 |
| Myt1 | myelin transcription factor 1 (Myt1), mRNA. | GI_23346428-S | 795 | 8.0 | **0.024** |
| Cidea | cell death-inducing DNA fragmentation factor, alpha subunit-like effector A (Cidea) | scl51827.6.1_1-S | 515 | 8.0 | **0.036** |
| A230058F20Rik | RIKEN cDNA A230058F20 gene | scl54973.1.1243_164-S | 376 | 8.0 | 0.129 |
| 1190002F15Rik | RIKEN cDNA 1190002F15 gene | scl0381822.1_19-S | 375 | 8.0 | 0.063 |
| B230216G23Rik | RIKEN cDNA B230216G23 gene | scl29383.4.1_215-S | 302 | 8.0 | **0.009** |
| Gad1 | glutamic acid decarboxylase 1 (Gad1), mRNA. | scl0003045.1_120-S | 199 | 8.0 | **0.040** |
| 1700023F06Rik | RIKEN cDNA 1700023F06 gene (1700023F06Rik), mRNA. | scl39486.4.1_56-S | 380 | 8.0 | **0.016** |
| Col9a2 | procollagen, type IX, alpha 2 (Col9a2), mRNA. | scl012840.32_211-S | 245 | 7.9 | **0.010** |
| N4bp2l1 | NEDD4 binding protein 2-like 1 (N4bp2l1), mRNA | scl070229.1_18-S | 296 | 7.9 | 0.113 |
| Lrg1 | leucine-rich alpha-2-glycoprotein 1 (Lrg1), mRNA. | scl49773.2_28-S | 1716 | 7.8 | **0.025** |
| **Syt7** | synaptotagmin VII | scl6307.1.1_179-S | 342 | 7.8 | **0.048** |
| Emb | embigin (Emb), mRNA. | scl013723.9_229-S | 1091 | 7.8 | **0.015** |

| | | | | | |
|---|---|---|---|---|---|
| Nrxn2 | neurexin II (Nrxn2), mRNA | scl52878.1.594_203-S | 335 | 7.8 | **0.032** |
| B3galt3 | UDP-Gal:betaGlcNAc beta 1,3-galactosyltransferase, polypeptide 3 (B3galt3), mRNA. | scl22075.4_6-S | 570 | 7.7 | **0.031** |
| D3Bwg0562e | DNA segment, Chr 3, Brigham & Womens Genetics 0562 expressed (D3Bwg0562e), mRNA. | scl21599.7_90-S | 216 | 7.7 | **0.002** |
| Svop | SV2 related protein (Svop), mRNA | scl26188.18_422-S | 384 | 7.7 | **0.029** |
| Mmp24 | matrix metalloproteinase 24 (Mmp24), mRNA. | scl20046.9_689-S | 341 | 7.6 | **0.011** |
| **Gria3** | glutamate receptor, ionotropic, AMPA3 (alpha 3) (Gria3), mRNA. | scl8260.1.1_298-S | 489 | 7.6 | 0.115 |
| Ica1l | islet cell autoantigen 1-like (Ica1l), mRNA | scl16712.12_256-S | 298 | 7.6 | **0.009** |
| **Neurod1** | neurogenic differentiation 1 (Neurod1), mRNA. | scl19085.2_285-S | 452 | 7.6 | **0.004** |
| Rasgrf1 | RAS protein-specific guanine nucleotide-releasing factor 1 (Rasgrf1), mRNA. | scl36632.28.1_170-S | 523 | 7.6 | 0.059 |
| Brsk2 | BR serine/threonine kinase 2 (Brsk2), mRNA | scl075770.2_292-S | 344 | 7.6 | **0.024** |
| C230072K23 | | scl0328429.2_283-S | 143 | 7.6 | **0.046** |
| **Syt1** | synaptotagmin 1 (Syt1), mRNA. | scl0020979.2_107-S | 314 | 7.5 | **0.002** |
| Rfx4 | regulatory factor X, 4 (influences HLA class II expression) (Rfx4), mRNA. | scl38612.22.1_93-S | 211 | 7.5 | 0.059 |
| Cyfip2 | cytoplasmic FMR1 interacting protein 2 (Cyfip2), mRNA. | scl40305.32_46-S | 698 | 7.5 | **0.025** |
| 5930404A08Rik | | ri\|5930404A08\|PX00055G02\|AK031091\|2291-S | 288 | 7.5 | **0.041** |
| **Syn1** | synapsin I (Syn1), mRNA. | scl54358.13_95-S | 524 | 7.5 | **0.001** |
| Eif2s3y | eukaryotic translation initiation factor 2, subunit 3, structural gene Y-linked (Eif2s3y), mRNA. | scl0026908.1_233-S | 186 | 7.3 | 0.407 |
| Cpe | carboxypeptidase E (Cpe), mRNA. | scl34760.11.1_14-S | 8314 | 7.3 | **0.008** |
| **Syngr3** | synaptogyrin 3 (Syngr3), mRNA. | scl50202.5_41-S | 200 | 7.3 | **0.008** |
| Nkx6-1 | NK6 transcription factor related, locus 1 (Drosophila) (Nkx6-1), mRNA. | scl26314.4.1_182-S | 220 | 7.2 | **0.001** |
| Gm885 | predicted gene 885 | GI_38091893-S | 254 | 7.2 | **0.008** |
| Ap3b2 | adaptor-related protein complex 3, beta 2 subunit (Ap3b2), mRNA. | scl31109.12.1_24-S | 206 | 7.2 | **0.010** |
| Smarca1 | SWI/SNF related, matrix associated, actin dependent regulator of chromatin, subfamily a, member 1 (Smarca1), mRNA. | scl54295.26.1_21-S | 680 | 7.2 | 0.051 |
| LOC333501 | similar to immunoglobulin kappa light chain variable region precursor (LOC333501), mRNA. | IGKV10-95_AF029261_Ig_kappa_variable_10-95_20-S | 419 | 7.0 | 0.209 |
| Olfm1 | olfactomedin 1 (Olfm1), mRNA. | scl0003387.1_1121-S | 554 | 7.0 | **0.010** |
| Gpr158 | G protein-coupled receptor 158 (Gpr158), mRNA | scl21222.12_521-S | 200 | 7.0 | 0.050 |
| Plcxd3 | phosphatidylinositol-specific phospholipase C, X domain containing 3 | scl48135.4.1_204-S | 175 | 7.0 | 0.064 |
| Rims3 | regulating synaptic membrane exocytosis 3 (Rims3), mRNA. | scl25010.7_694-S | 229 | 7.0 | 0.050 |
| Slp | sex-limited protein (Slp), mRNA. | scl0020567.1_162-S | 1136 | 7.0 | **0.005** |
| Igkv12-98 | immunoglobulin kappa variable 12-98 | IGKV12-98_AJ235949_Ig_kappa_variable_12-98_12-S | 182 | 7.0 | 0.309 |
| Lin7a | lin 7 homolog a (C. elegans) (Lin7a), mRNA. | scl00103301.1_320-S | 247 | 7.0 | **0.019** |
| Rab3a | RAB3A, member RAS oncogene family (Rab3a), mRNA. | scl019339.5_272-S | 453 | 6.9 | **0.009** |

| | | | | | |
|---|---|---|---|---|---|
| Snap91 | synaptosomal-associated protein 91 (Snap91), mRNA. | scl020616.1_75-S | 149 | 6.8 | **0.005** |
| Gpr85 | G protein-coupled receptor 85 (Gpr85), mRNA. | scl0064450.1_199-S | 614 | 6.8 | **0.007** |
| Ust | uronyl-2-sulfotransferase (Ust), mRNA | scl38227.11_29-S | 484 | 6.7 | 0.053 |
| N4bp2l1 | NEDD4 binding protein 2-like 1 ( N4bp2l1), mRNA. | scl0100637.1_115-S | 752 | 6.7 | 0.072 |
| Add2 | adducin 2 (beta) (Add2), mRNA | scl072970.1_11-S | 180 | 6.7 | **0.040** |
| St6gal1 | beta galactoside alpha 2,6 sialyltransferase 1 (St6gal1), mRNA. | scl49300.12_501-S | 2023 | 6.7 | **0.017** |
| Stc1 | stanniocalcin 1 (Stc1), mRNA. | scl46141.4_0-S | 390 | 6.7 | **0.031** |
| Unc80 | unc-80 homolog (C. elegans) (Unc80), mRNA | scl14607.1.1_266-S | 188 | 6.7 | **0.046** |
| BC007180 | | scl22435.1.2_91-S | 262 | 6.6 | **0.037** |
| 5330439M10Rik | RIKEN cDNA 5330439M10 gene (5330439M10Rik), mRNA. | scl38395.1.7_56-S | 205 | 6.6 | 0.095 |
| Cntnap2 | contactin associated protein-like 2 (Cntnap2), mRNA. | scl066797.3_51-S | 219 | 6.6 | **0.013** |
| **Syt5** | synaptotagmin 5 (Syt5), mRNA. | scl31809.10.1_30-S | 171 | 6.6 | **0.004** |
| Pnma2 | paraneoplastic antigen MA2 (Pnma2), mRNA. | scl46152.5_553-S | 257 | 6.6 | **0.000** |
| Prrt2 | proline-rich transmembrane protein 2 (Prrt2), mRNA | scl069017.6_7-S | 220 | 6.6 | **0.041** |
| C030014C12Rik | | scl43324.1.1_319-S | 243 | 6.6 | 0.087 |
| Nrip3 | nuclear receptor interacting protein 3 (Nrip3), mRNA. | scl30829.8_606-S | 290 | 6.5 | **0.001** |
| C4 | complement component 4 (within H-2S) (C4), mRNA. | scl012268.2_49-S | 354 | 6.5 | **0.004** |
| Sept3 | septin 3 (Sept3), mRNA | scl078356.1_225-S | 262 | 6.5 | **0.008** |
| 2900011O08Rik | RIKEN cDNA 2900011O08 gene (2900011O08Rik), mRNA. | scl49400.6_138-S | 211 | 6.5 | **0.007** |
| Nol4 | nucleolar protein 4 (Nol4), mRNA. | scl0319211.1_216-S | 288 | 6.5 | **0.021** |
| Car10 | carbonic anhydrase 10 (Car10), mRNA. | scl41039.12.1_215-S | 168 | 6.5 | 0.068 |
| Col8a1 | procollagen, type VIII, alpha 1 (Col8a1), mRNA. | scl490.1.1_278-S | 219 | 6.5 | 0.055 |
| Amph | amphiphysin (Amph), mRNA. | scl45034.23_392-S | 406 | 6.5 | **0.032** |
| Gdap1 | ganglioside-induced differentiation-associated-protein 1 (Gdap1), mRNA. | scl0014545.2_19-S | 236 | 6.5 | **0.019** |
| Gnao1 | guanine nucleotide binding protein, alpha O (Gnao1), mRNA | scl33497.11_25-S | 1148 | 6.5 | **0.049** |
| Ggtl3 | gamma-glutamyltransferase-like 3 (Ggtl3), mRNA. | scl18460.16.1_0-S | 227 | 6.5 | **0.013** |
| Kcnc1 | potassium voltage gated channel, Shaw-related subfamily, member 1 (Kcnc1), mRNA | scl0320399.1_192-S | 162 | 6.5 | **0.024** |
| Pkib | protein kinase inhibitor beta, cAMP dependent, testis specific | ri\|1500017O11\|ZX00057K11\|AK005281\|1288-S | 294 | 6.4 | **0.035** |
| Pap | pancreatitis-associated protein (Pap), mRNA. | scl29870.6.1_39-S | 296 | 6.4 | 0.187 |
| Cxcl2 | chemokine (C-X-C motif) ligand 2 (Cxcl2), mRNA. | scl020310.4_115-S | 170 | 6.4 | 0.134 |
| Tmem22 | transmembrane protein 22 | GI_38090024-S | 882 | 6.4 | **0.011** |
| 2700031B12Rik | | ri\|2700031B12\|ZX00063G16\|AK012306\|1196-S | 254 | 6.4 | 0.081 |
| Iapp | islet amyloid polypeptide (Iapp), mRNA. | scl29386.3.1_1-S | 351 | 6.4 | 0.174 |
| Zcchc12 | zinc finger, CCHC domain containing 12 (Zcchc12), mRNA | scl54999.4_50-S | 482 | 6.4 | **0.004** |
| Fam171b | family with sequence similarity 171, member B | scl00241520.2_184-S | 344 | 6.4 | **0.010** |
| Cdh22 | cadherin 22 (Cdh22), mRNA. | scl18336.12.1_69-S | 119 | 6.3 | **0.025** |
| Fam131a | family with sequence similarity 131, member A (Fam131a), mRNA | scl078408.1_245-S | 429 | 6.3 | **0.024** |
| Mamdc1 | MAM domain containing 1 (Mamdc1), mRNA. | scl00320772.1_270-S | 233 | 6.2 | **0.039** |

| | | | | | |
|---|---|---|---|---|---|
| Cntnap2 | contactin associated protein-like 2 (Cntnap2), mRNA. | scl0066797.1_308-S | 281 | 6.2 | 0.064 |
| Gtl2 | GTL2, imprinted maternally expressed untranslated mRNA (Gtl2), mRNA. | scl42789.1.791_155-S | 223 | 6.2 | 0.058 |
| Dos | downstream of Stk11 (Dos), mRNA. | scl0216164.1_206-S | 801 | 6.2 | **0.036** |
| Gng4 | guanine nucleotide binding protein (G protein), gamma 4 subunit (Gng4), mRNA. | scl45060.6_526-S | 509 | 6.2 | **0.000** |
| Fev | FEV (ETS oncogene family) (Fev), mRNA. | scl00260298.2_181-S | 182 | 6.2 | **0.001** |
| **Ddc** | dopa decarboxylase | ri\|2610109O21\|ZX00061I24\|AK011834\|1441-S | 256 | 6.1 | **0.021** |
| Mmp13 | matrix metalloproteinase 13 (Mmp13), mRNA. | scl37309.10_320-S | 486 | 6.0 | **0.024** |
| Tuba1 | tubulin, alpha 1 (Tuba1), mRNA. | scl022142.1_236-S | 668 | 6.0 | **0.012** |
| Rtn1 | reticulon 1 (Rtn1), mRNA. | scl00104001.2_121-S | 360 | 6.0 | **0.011** |
| Serp2 | stress-associated endoplasmic reticulum protein family member 2 (Serp2), mRNA | scl45288.5.3_74-S | 238 | 6.0 | **0.018** |
| 2610042L04Rik | RIKEN cDNA 2610042L04 gene (2610042L04Rik), mRNA. | scl0067055.1_222-S | 96 | 5.9 | **0.039** |
| Nap1l5 | nucleosome assembly protein 1-like 5 (Nap1l5), mRNA. | scl28956.1_12-S | 250 | 5.9 | **0.021** |
| Abcb4 | ATP-binding cassette, sub-family B (MDR/TAP), member 4 (Abcb4), mRNA. | scl26912.28.1_161-S | 122 | 5.9 | **0.003** |
| Dnajc12 | DnaJ (Hsp40) homolog, subfamily C, member 12 (Dnajc12), mRNA. | scl38846.4.1_6-S | 773 | 5.9 | **0.024** |
| 6430537F04 | | scl24334.3_574-S | 213 | 5.9 | **0.033** |
| Gsdma | gasdermin A (Gadma), mRNA | scl057911.12_253-S | 337 | 5.9 | **0.035** |
| Mtap1b | microtubule-associated protein 1 B (Mtap1b), mRNA. | scl017755.1_80-S | 330 | 5.8 | **0.015** |
| Isp1 | implantation serine protease 1 (Isp1), mRNA. | scl50985.6.1_72-S | 184 | 5.8 | 0.260 |
| Aatk | apoptosis-associated tyrosine kinase (Aatk), mRNA. | scl39250.15_28-S | 1113 | 5.8 | **0.014** |
| Rmst | rhabdomyosarcoma 2 associated transcript (non-coding RNA) | scl0320034.2_90-S | 252 | 5.8 | 0.072 |
| Bhlhb9 | basic helix-loop-helix domain containing, class B9 (Bhlhb9), mRNA | scl54620.5_512-S | 897 | 5.8 | **0.007** |
| Gm1631 | gene model 1631, (NCBI) (Gm1631), mRNA. | scl0381371.3_13-S | 178 | 5.7 | **0.048** |
| Mt3 | metallothionein 3 (Mt3), mRNA. | scl017751.1_8-S | 228 | 5.7 | **0.001** |
| 5330431K02Rik | RIKEN cDNA 5330431K02 gene (5330431K02Rik), mRNA. | scl43611.1.1_320-S | 493 | 5.7 | **0.032** |
| Tubg2 | tubulin, gamma 2 (Tubg2), mRNA. | scl40892.11.1_309-S | 248 | 5.7 | **0.028** |
| Gsbs | G substrate (Gsbs), mRNA. | scl30000.5_56-S | 169 | 5.7 | 0.119 |
| Lcn2 | lipocalin 2 (Lcn2), mRNA. | scl19446.4_4-S | 248 | 5.7 | **0.001** |
| Igkv4-77 | immunoglobulin kappa variable 4-77 | IGKV4-77_AJ235940_Ig_kappa_variable_4-77_18-S | 205 | 5.6 | 0.244 |
| Gm1673 | predicted gene 1673 | GI_38079650-S | 432 | 5.6 | **0.047** |
| Mmp10 | matrix metalloproteinase 10 (Mmp10), mRNA. | scl37305.10.1_6-S | 731 | 5.5 | **0.038** |
| Syt11 | synaptotagmin 11 (Syt11), mRNA. | scl21963.5_3-S | 1710 | 5.5 | **0.010** |
| Serping1 | serine (or cysteine) proteinase inhibitor, clade G, member 1 (Serping1), mRNA. | scl19066.9.1_77-S | 3904 | 5.5 | **0.013** |
| Spnb1 | spectrin beta 1 (Spnb1), mRNA | scl0020741.1_267-S | 239 | 5.5 | **0.003** |
| Riiad1 | regulatory subunit of type II PKA R-subunit (RIIa) domain containing 1 (Riiad1), mRNA | scl21867.7.1_21-S | 391 | 5.5 | **0.007** |
| Fez1 | fasciculation and elongation protein zeta 1 (zygin I) (Fez1), mRNA. | scl37121.13.1_3-S | 383 | 5.5 | **0.018** |
| Sox2 | SRY-box containing gene 2 (Sox2), mRNA. | scl020674.7_60-S | 193 | 5.5 | **0.000** |

| | | | | | |
|---|---|---|---|---|---|
| Pgbd5 | piggyBac transposable element derived 5 (Pgbd5), mRNA | scl067257.1_121-S | 316 | 5.5 | **0.028** |
| Pcdhb4 | protocadherin beta 4 (Pcdhb4), mRNA. | scl52071.1.29_20-S | 148 | 5.5 | **0.032** |
| Cacna2d1 | calcium channel, voltage-dependent, alpha2/delta subunit 1 | ri\|D830029A14\|PX00199P04\|AK052898\|1271-S | 358 | 5.5 | **0.041** |
| Camk2n1 | calcium/calmodulin-dependent protein kinase II inhibitor 1 | scl066259.1_113-S | 1694 | 5.5 | **0.009** |
| Mmp3 | matrix metalloproteinase 3 (Mmp3), mRNA. | scl37307.8.1_29-S | 256 | 5.5 | 0.060 |
| Slp | sex-limited protein (Slp), mRNA. | scl020567.3_14-S | 1015 | 5.4 | **0.021** |
| Cacng2 | calcium channel, voltage-dependent, gamma subunit 2 (Cacng2), mRNA. | scl46997.4_329-S | 156 | 5.4 | **0.016** |
| Ina | internexin neuronal intermediate filament protein, alpha (Ina), mRNA. | GI_34328367-S | 130 | 5.4 | **0.013** |
| 2700050C19Rik | RIKEN cDNA 1500009L16 gene | scl070232.4_204-S | 234 | 5.4 | **0.012** |
| D130083G05Rik | | ri\|D130083G05\|PX00186P02\|AK084071\|1297-S | 524 | 5.4 | 0.067 |
| Ppp2r2c | protein phosphatase 2 (formerly 2A), regulatory subunit B (PR 52), gamma isoform | scl27887.10_414-S | 210 | 5.4 | **0.000** |
| Il1b | interleukin 1 beta (Il1b), mRNA. | scl18674.7.1_35-S | 316 | 5.4 | 0.230 |
| Cdh13 | cadherin 13 (Cdh13), mRNA. | scl0012554.2_76-S | 602 | 5.3 | **0.015** |
| Tecta | tectorin alpha (Tecta), mRNA. | scl35982.24.1_47-S | 187 | 5.3 | **0.033** |
| Ppp1r3f | protein phosphatase 1, regulatory (inhibitor) subunit 3F (Ppp1r3f), mRNA. | scl54449.5_574-S | 268 | 5.3 | **0.023** |
| Stmn1 | stathmin 1 (Stmn1), mRNA. | scl016765.5_129-S | 720 | 5.3 | 0.058 |
| Smyd4 | SET and MYND domain containing 4 (Smyd4), mRNA. | scl41266.11.1_55-S | 273 | 5.3 | **0.018** |
| Tceal5 | Tceal5, transcription elongation factor A (SII)-like 5 (Tceal5), mRNA | scl53816.4_374-S | 511 | 5.3 | **0.010** |
| Igl-V1 | immunoglobulin lambda variable 1 | scl016142.1_30-S | 464 | 5.3 | 0.225 |
| **Sv2b** | synaptic vesicle glycoprotein 2 b (Sv2b), mRNA. | scl31171.16_606-S | 333 | 5.3 | **0.039** |
| Clgn | calmegin (Clgn), mRNA. | scl33608.15_606-S | 240 | 5.3 | 0.051 |
| Cckbr | cholecystokinin B receptor (Cckbr), mRNA. | scl012426.5_191-S | 617 | 5.3 | **0.006** |
| LOC328644 | hypothetical gene supported by AK045595 (LOC328644), mRNA. | scl0328644.3_317-S | 218 | 5.2 | 0.060 |
| Slc35f3 | solute carrier family 35, member F3 (Slc35f3), mRNA. | scl33165.11.1_4-S | 183 | 5.2 | **0.011** |
| Prkcb | protein kinase C, beta (Prkcb), mRNA | scl018751.17_49-S | 622 | 5.2 | **0.011** |
| D630047N04Rik | neurotransmitter transporter (fragment) homolog [Mus musculus] | ri\|D630047N04\|PX00198K07\|AK085620\|3080-S | 122 | 5.2 | 0.054 |
| Nsg1 | neuron specific gene family member 1 (Nsg1), mRNA. | scl26668.5_235-S | 210 | 5.2 | **0.001** |
| Diras2 | DIRAS family, GTP-binding RAS-like 2 (Diras2), mRNA | scl43971.2.2_53-S | 1126 | 5.2 | **0.024** |
| Fez1 | fasciculation and elongation protein zeta 1 (zygin I) (Fez1), mRNA. | scl0003544.1_55-S | 419 | 5.2 | **0.008** |
| LOC385615 | similar to hypothetical protein FLJ10201 (LOC385615), mRNA. | GI_38080559-S | 131 | 5.1 | 0.052 |
| Myo5a | myosin VA | scl36699.4_279-S | 274 | 5.1 | **0.012** |
| Fbxl16 | F-box and leucine-rich repeat protein 16 | scl0214931.6_73-S | 157 | 5.1 | **0.000** |
| NCAM-140 | Mouse mRNA fragment for neural cell adhesion molecule (NCAM-140). | scl0003519.1_175-S | 749 | 5.1 | 0.057 |
| LOC243430 | similar to Ig kappa light chain precursor (LOC243430), mRNA. | IGKV9-124_AF003294_Ig_kappa_variable_9-124_18-S | 347 | 5.1 | 0.129 |
| Fscn1 | fascin homolog 1, actin bundling protein (Strongylocentrotus) purpuratus) (Fscn1), mRNA. | scl27024.7_349-S | 328 | 5.1 | **0.004** |

| | | | | | |
|---|---|---|---|---|---|
| Tm4sf4 | transmembrane 4 superfamily member 4 (Tm4sf4), mRNA. | scl23175.3_26-S | 1245 | 5.0 | **0.012** |
| 6330509M05Rik | RIKEN cDNA 6330509M05 gene | scl7210.1.1_253-S | 451 | 5.0 | **0.024** |
| Igkv4-73 | immunoglobulin kappa variable 4-73 | IGKV4-73_AJ231216_Ig_kappa_variable_4-73_18-S | 407 | 5.0 | 0.210 |
| Eef1a2 | eukaryotic translation elongation factor 1 alpha 2 (Eef1a2), mRNA. | scl18213.3.5_8-S | 101 | 5.0 | **0.003** |
| Ndn | necdin (Ndn), mRNA. | scl32592.2.658_11-S | 2985 | 5.0 | **0.007** |
| Opcml | opioid binding protein/cell adhesion molecule-like (Opcml), mRNA | scl37169.1_27-S | 199 | 5.0 | **0.038** |
| Rundc3b, | RUN domain containing 3B | scl0242819.10_30-S | 205 | 5.0 | **0.012** |
| Mllt11 | myeloid/lymphoid or mixed-lineage leukemia (trithorax homolog, Drosophila); translocated to, 11 | scl056772.1_0-S | 896 | 5.0 | **0.000** |
| Fam163a | family with sequence similarity 163, member A | scl16107.5.1_203-S | 122 | 5.0 | **0.000** |
| **Ncam1** | neural cell adhesion molecule 1 (Ncam1), mRNA. | scl35883.23_148-S | 466 | 5.0 | **0.018** |
| Zfp365 | zinc finger protein 365 (Zfp365), mRNA. | scl37856.7_341-S | 226 | 5.0 | **0.019** |

[1] genes encoding known neuroendocrine proteins or transcription factors involved the development of the neuroendocrine lineage are shown in bold; [2] p values < 0.05 are shown in bold
