# Supplementary material for: Expression of a Neuroendocrine Gene Signature in Gastric Tumor Cells from CEA 424-SV40 Large T Antigen-Transgenic Mice Depends on SV40 Large T Antigen
Source: PLoS One. 2012 Jan 13;7(1):e29846. doi: 10.1371/journal.pone.0029846 (PMC3258231; doi:10.1371/journal.pone.0029846)
Supplement: Table S2 — Fraction of neuroendocrine (NE) genes/probe sets up- or down-regulated in tumors of CEA424-SV40 TAg mice. (PDF) [file pone.0029846.s003.pdf]

Table S2: Fraction of neuroendocrine (NE) genes/probe sets up- or down-regulated in tumors of *CEA424*-SV40 TAg mice: A gene was classified as “neuroendocrine gene” when ≥ 10 publications were identified with the search terms “<long form of gene name>” and “neuroendocrine” in PubMed (October 2011).

**Age of tumor-bearing mouse**	**Fold overexpression in tumor-bearing vs normal antrum (upregulated in tumor)**	**Fraction of NE genes (overexpressed NE / total overexpressed genes)**
30 d	≥ 20	- % (0/0)
30 d	≥ 10	50 % (1/2)
30 d	≥ 5	75 % (3/4)
60 d	≥ 20	100 % (2/2)
60 d	≥ 10	86 % (6/7)
60 d	≥ 5	30 % (8/27)
90 d	≥ 20	38 % (16/42)
90 d	≥ 10	20 % (26/128)
90 d	≥ 5	11 % (38/338)
**Age of tumor-bearing mouse**	**Fold overexpression in normal vs tumor-bearing antrum (downregulated in tumor)**	**Fraction of NE genes (downregulated NE / total downregulated genes)**
90 d	≥ 5	1.6 % (2*/129)

*secretin, gastrin
