# Supplementary material for: Expression of a Neuroendocrine Gene Signature in Gastric Tumor Cells from CEA 424-SV40 Large T Antigen-Transgenic Mice Depends on SV40 Large T Antigen
Source: PLoS One. 2012 Jan 13;7(1):e29846. doi: 10.1371/journal.pone.0029846 (PMC3258231; doi:10.1371/journal.pone.0029846)
Supplement: Table S3 — Expression of transcription factor and DNA-binding protein genes from the Syder signature in d90 CEA424-SV40 TAg tumors. (PDF) [file pone.0029846.s004.pdf]

Table S3: Expression of transcription factor and DNA-binding protein genes from the Syder signature in d90 *CEA424*-SV40 TAg tumors

Gene	Description	Accession	Probe set[1]	fluorescence d90 tumor	fold change	P value[2]
Nkx2-2	NK2 transcription factor related, locus 2 (Drosophila) (Nkx2-2)	NM_010919.1	scl0018088.2_84-S	490	21.1	**0.010**
Etv1	ets variant gene 1 (Etv1)	NM_007960.1	scl014009.13_0-S	2437	17.3	**0.014**
Peg3	paternally expressed 3 (Peg3)	NM_008817	scl018616.1_273-S	851	11.9	0.077
Neurod1	neurogenic differentiation 1 (Neurod1)	NM_010894.1	scl19085.2_285-S	452	7.6	**0.004**
Sox2	SRY-box containing gene 2 (Sox2)	NM_011443.2	scl020674.7_60-S	193	5.5	**0.000**
Ndn	necdin (Ndn)	NM_010882.2	scl32592.2.658_11-S	2985	5.0	**0.007**
Cntn1	contactin 1 (Cntn1)	NM_007727	scl47598.31_288-S	193	4.9	**0.029**
Peg3	paternally expressed 3 (Peg3)	NM_008817.1	scl0018616.2_167-S	134	4.8	**0.040**
Hey1	hairy/enhancer-of-split related with YRPW motif 1 (Hey1)	NM_010423.1	scl22405.4_235-S	241	3.3	**0.003**
Myef2	myelin basic protein expression factor 2, repressor (Myef2)	NM_010852.1	scl0017876.1_262-S	225	3.0	**0.020**
Crem	cAMP responsive element modulator (Crem)	NM_013498.1	scl51699.17_197-S	135	2.9	**0.010**
Ddef1	development and differentiation enhancing (Ddef1)	NM_010026.1	scl47133.29_179-S	267	2.4	**0.001**
Zfp612	zinc finger protein 612 (Zfp612), mRNA.	NM_175480.3	scl00234725.2_22-S	193	2.4	**0.009**
Sox2	SRY-box containing gene 2 (Sox2)	NM_011443.2	scl23279.1.1_50-S	136	2.4	**0.000**
Nap1l1	nucleosome assembly protein 1-like 1 (Nap1l1)	NM_015781.2	scl38446.16_40-S	352	2.4	**0.001**
Atf5	activating transcription factor 5 (Atf5)	NM_030693.1	scl31408.4_131-S	640	2.2	**0.022**
Cugbp2	CUG triplet repeat,RNA binding protein 2 (Cugbp2)	NM_010160.1	scl014007.1_13-S	269	2.1	**0.000**
Ppargc1a	peroxisome proliferative activated receptor, gamma, coactivator 1 alpha (Ppargc1a)	NM_008904.1	scl26595.14.1_90-S	229	1.7	**0.036**
Cugbp2	CUG triplet repeat,RNA binding protein 2 (Cugbp2)	NM_010160.1	scl0014007.1_233-S	345	1.5	**0.008**
Nap1l1	nucleosome assembly protein 1-like 1 (Nap1l1)	NM_015781.2	scl0003917.1_104-S	569	1.5	**0.031**
Ssbp2	single-stranded DNA binding protein 2 (Ssbp2)	NM_024186.1	scl0066970.1_29-S	119	1.5	**0.031**
Ssbp2	single-stranded DNA binding protein 2 (Ssbp2)	NM_024186.1	scl0003713.1_190-S	242	1.4	**0.036**
Rbm18	RNA binding motif protein 18 (Rbm18), mRNA.	NM_026434.2	scl19388.5_90-S	410	1.3	0.058
Nr1d2	nuclear receptor subfamily 1, group D, member 2 (Nr1d2)	NM_011584.2	scl00353187.1_48-S	163	1.2	0.174
Zfhx1a/Zeb1	zinc finger homeobox 1a (Zfhx1a)	NM_011546.1	scl0021417.2_96-S	211	1.1	0.421
Nr2f1	nuclear receptor subfamily 2, group F, member 1 (Nr2f1)	NM_010151.1	scl43736.6_95-S	282	-1.2	0.391
Irf1	interferon regulatory factor 1 (Irf1)	NM_008390.1	scl0001336.1_35-S	187	-1.4	0.375
Hopx	homeobox only domain (Hod), mRNA.	AK003784	ri|1110018K11|R00001	359	-1.5	0.378

[1] only probe sets exhibiting a fluorescence of >100 (RFU) are shown; [2] p values < 0.05 are shown in bold
