# Supplementary material for: Expression of a Neuroendocrine Gene Signature in Gastric Tumor Cells from CEA 424-SV40 Large T Antigen-Transgenic Mice Depends on SV40 Large T Antigen
Source: PLoS One. 2012 Jan 13;7(1):e29846. doi: 10.1371/journal.pone.0029846 (PMC3258231; doi:10.1371/journal.pone.0029846)
Supplement: Table S4 — Genes deregulated in 424GC cells by SV40 TAg-specific siRNA. (PDF) [file pone.0029846.s005.pdf]

Supplementary Table S4: Genes deregulated in 424GC cells by SV40 TAg-specific siRNA

Gene[1]	Description	Probe set	Expression (mean) without TAg siRNA	Fold down-regulation +siRNA	P value[2]
Mela	melanoma antigen	scl017276.1_218-S	1440	7.49	**0.000**
MSV	Murine sarcoma virus	9626100_15-S	4360	5.27	**0.000**
mt-Nd4l	mt-Nd4l	scl0017720.1_306-S	1365	4.86	**0.000**
Tmed2	transmembrane emp24 domain trafficking protein 2	scl056334.4_3-S	2219	4.65	**0.000**
Hnrpa2b1	heterogeneous nuclear ribonucleoprotein A2/B1	scl0053379.2_22-S	1396	4.56	**0.000**
Abl-MuLV	Abelson murine leukemia virus	9626953_200-S	2238	4.10	**0.000**
Eif4g2	eukaryotic translation initiation factor 4, gamma 2	scl000210.1_44-S	562	4.09	**0.000**
Ldha	lactate dehydrogenase A	scl0001816.1_0-S	1179	3.90	**0.000**
Ldha	lactate dehydrogenase A	scl016828.6_330-S	2367	3.88	**0.000**
Snurf	SNRPN upstream reading frame	scl000252.1_5-S	1124	3.77	**0.000**
Rbbp7	retinoblastoma binding protein 7	scl0245688.1_74-S	682	3.54	**0.000**
Tubb5	tubulin, beta 5	scl49975.4.297_19-S	856	3.54	**0.000**
**Gnas**	GNAS (guanine nucleotide binding protein, alpha stimulating) complex locus	scl014683.1_3-S	1884	3.53	**0.000**
Ppp1cb	protein phosphatase 1, catalytic subunit, beta isoform	scl0004044.1_9-S	538	3.48	**0.000**
Tcp1	t-complex protein 1	scl0001621.1_13-S	602	3.48	**0.000**
NA	RIKEN cDNA 1810009A16 gene	scl0002667.1_44-S	396	3.42	**0.000**
Psmd13	proteasome (prosome, macropain) 26S subunit, non-ATPase, 13	scl00071.1_7-S	777	3.41	**0.000**
**Pdyn**	prodynorphin	scl18671.5_48-S	5796	3.34	**0.000**
Snrpn	small nuclear ribonucleoprotein N	scl0020646.1_10-S	764	3.33	**0.000**
Ppp1ca	protein phosphatase 1, catalytic subunit, alpha isoform	scl000534.1_21-S	1644	3.30	**0.000**
Ccnb1	cyclin B1	scl0268697.4_6-S	1814	3.29	**0.000**
NA	similar to G2/mitotic-specific cyclin B1 (LOC328752)	GI_38075552-S	435	3.17	**0.000**
Pgk1	phosphoglycerate kinase 1	scl018655.12_25-S	7134	3.16	**0.000**
NA	hypothetical protein LOC280487	GI_38081456-I	404	3.14	**0.000**
Tpx2	TPX2, microtubule-associated protein homolog (Xenopus laevis)	scl20105.20_178-S	1215	3.06	**0.000**
Emp1	epithelial membrane protein 1	scl29429.7_143-S	1718	3.04	**0.000**
Tpm3	tropomyosin 3, gamma	scl0001950.1_6-S	908	3.02	**0.000**
**Chga**	chromogranin A	scl0002400.1_38-S	6386	2.99	**0.000**
NA	expressed sequence AI505012	scl46834.6.2176_154-S	654	2.99	**0.000**
Atp5k	ATP synthase, H+ transporting, mitochondrial F1F0 complex, subunit e	scl011958.2_29-S	1378	2.98	**0.000**
3110032G18Rik	RIKEN cDNA 3110032G18 gene	scl27213.3_538-S	1351	2.97	**0.000**

Tram1	translocating chain-associating membrane protein 1	scl16982.5.1_3-S	481	2.97	**0.000**
Cdkn2c	cyclin-dependent kinase inhibitor 2C (p18, inhibits CDK4)	scl0002624.1_576-S	725	2.95	**0.000**
Mki67	antigen identified by monoclonal antibody Ki 67	scl0017345.1_1-S	1349	2.92	**0.000**
Fkbp11	FK506 binding protein 11	scl46759.5.1_10-S	1425	2.91	**0.000**
Kns2	kinesin 2	scl0002405.1_260-S	822	2.89	**0.000**
Cltc	clathrin, heavy polypeptide	scl0001345.1_85-S	649	2.89	**0.000**
Rbbp7	retinoblastoma binding protein 7	scl00245688.1_91-S	631	2.89	**0.000**
Pmp22	peripheral myelin protein	scl018858.5_31-S	717	2.88	**0.000**
Ccnb1	cyclin B1	scl43586.8_295-S	1264	2.87	**0.000**
NA	similar to RIKEN cDNA 2610524H06 gene	GI_38079875-S	556	2.86	**0.000**
Pcna	proliferating cell nuclear antigen	scl018538.1_192-S	1782	2.86	**0.000**
Fin14	fibroblast growth factor inducible 14	scl28579.2_420-S	1074	2.85	**0.000**
Arl5a	ADP-ribosylation factor-like 5A	scl0075423.2_59-S	354	2.83	**0.000**
Atp5l	similar to ATP synthase, H+ transporting, mitochondrial F0 complex,	GI_20888180-I	1946	2.83	**0.000**
Ndufa5	NADH dehydrogenase (ubiquinone) 1 alpha subcomplex, 5	scl0001121.1_1-S	1511	2.82	**0.000**
Hnrph1	heterogeneous nuclear ribonucleoprotein H1	scl059013.8_10-S	1307	2.80	**0.000**
Tmed2	transmembrane emp24 domain trafficking protein 2	scl0056334.1_201-S	757	2.80	**0.000**
Mcm5	minichromosome maintenance deficient 5, cell division cycle 46 (S. cerevisiae)	scl33659.17.1_21-S	2655	2.77	**0.000**
Mo-MSV	Moloney murine sarcoma virus	9626962_229-S	529	2.77	**0.000**
Txndc14	thioredoxin domain containing 14	scl0003075.1_15-S	380	2.76	**0.000**
Dusp4	dual specificity phosphatase 4	scl33912.4_501-S	559	2.74	**0.000**
Kns2	kinesin 2	scl0016593.2_91-S	832	2.74	**0.000**
NA	similar to envelope polyprotein	GI_38081137-S	494	2.74	**0.000**
Stmn1	stathmin 1	scl016765.5_129-S	1995	2.73	**0.000**
Tnfrsf22	tumor necrosis factor receptor superfamily, member 22	scl0079202.2_327-S	606	2.72	**0.000**
Cdr2	cerebellar degeneration-related 2	scl30730.6_511-S	741	2.70	**0.000**
Tmem66	transmembrane protein 66	scl000679.1_109-S	655	2.70	**0.000**
Myef2	myelin basic protein expression factor 2, repressor	scl0017876.1_262-S	462	2.69	**0.000**
mtDNA_ND2-S	mtDNA_ND2-S	mtDNA_ND2-S	13103	2.69	**0.000**
Rauscher-MuLV	Rauscher murine leukemia virus	9629514_325-S	405	2.65	**0.000**
Cbx3	chromobox homolog 3 (Drosophila HP1 gamma)	scl012417.4_32-S	990	2.63	**0.000**
mtDNA_ND4L-S	mtDNA_ND4L-S	mtDNA_ND4L-S	5761	2.63	**0.000**
Rhoa	ras homolog gene family, member A	scl011848.5_0-S	1433	2.63	**0.000**
Hnrph1	heterogeneous nuclear ribonucleoprotein H1	scl0059013.2_121-S	689	2.62	**0.000**
Ucp2	uncoupling protein 2 (mitochondrial, proton carrier)	scl00035.1_45-S	2204	2.62	**0.000**
Mela	melanoma antigen	scl0017276.1_44-S	692	2.61	**0.000**

Cdc2a	cell division cycle 2 homolog A (S. pombe)	scl0003783.1_100-S	1156	2.60	**0.000**
Tyms	thymidylate synthase	scl0004125.1_0-S	823	2.60	**0.000**
Gata2	GATA binding protein 2	scl29775.9_427-S	895	2.59	**0.000**
Friend-MuLV	Friend murine leukemia virus	9626096_327-S	353	2.59	**0.000**
Cnot1	CCR4-NOT transcription complex, subunit 1	scl00234594.1_78-S	302	2.57	**0.000**
Eif4g2	eukaryotic translation initiation factor 4, gamma 2	scl013690.1_50-S	656	2.57	**0.000**
Top2a	topoisomerase (DNA) II alpha	scl39614.33.3_6-S	766	2.55	**0.000**
Clspn	claspin homolog (Xenopus laevis)	scl00269582.1_78-S	562	2.54	**0.000**
Ddost	dolichyl-di-phosphooligosaccharide-protein glycotransferase	scl24791.10_1-S	2372	2.54	**0.000**
Gpsn2	glycoprotein, synaptic 2	scl000669.1_31-S	2041	2.54	**0.000**
Kctd12	potassium channel tetramerisation domain containing 12	scl00239217.2_149-S	1084	2.53	**0.000**
3000004C01Rik	RIKEN cDNA 3000004C01 gene	scl0070218.1_56-S	526	2.49	**0.000**
Rer1	RER1 retention in endoplasmic reticulum 1 homolog (S. cerevisiae)	scl0002748.1_8-S	1464	2.48	**0.000**
NA	similar to glyceraldehyde-3-phosphate dehydrogenase	scl0277333.1_280-S	824	2.47	**0.000**
Dnajb11	DnaJ (Hsp40) homolog, subfamily B, member 11	scl49316.10.1_12-S	1829	2.47	**0.000**
Rad51ap1	RAD51 associated protein 1	scl00112420.1_5-S	382	2.47	**0.000**
Anapc5	anaphase-promoting complex subunit 5	scl0004124.1_11-S	279	2.46	**0.000**
1810047C23Rik	RIKEN cDNA 1810047C23 gene	scl000603.1_82-S	824	2.45	**0.000**
Ergic3	ERGIC and golgi 3	scl20039.1_29-S	473	2.45	**0.000**
NA	similar to RIKEN cDNA 2610524H06 gene	GI_38090371-S	253	2.45	**0.000**
S100a1	S100 calcium binding protein A1	scl21916.1_21-S	970	2.45	**0.000**
Ppapdc1	phosphatidic acid phosphatase type 2 domain containing 1	scl33962.6.1_87-S	408	2.44	**0.000**
NA	similar to Ab2-450	GI_38082567-S	376	2.43	**0.000**
NA	similar to CG13990-PA	GI_38095518-S	233	2.42	**0.000**
Papss2	3'-phosphoadenosine 5'-phosphosulfate synthase 2	scl0023972.1_63-S	420	2.41	**0.000**
Snrpa	small nuclear ribonucleoprotein polypeptide A	scl053607.7_2-S	261	2.41	**0.000**
2610036L11Rik	RIKEN cDNA 2610036L11 gene	ri|1700015C21|ZX00037I13|AK005988|408-S	1334	2.40	**0.000**
Nup85	nucleoporin 85	ri|5730441M17|PX00003D10|AK017632|2185-S	1596	2.40	**0.000**
NA	similar to RIKEN cDNA 2610524H06 gene	GI_38079905-S	411	2.39	**0.000**
NA	similar to CG32656-PA	GI_38093896-S	249	2.39	**0.000**
Cdkn3	cyclin-dependent kinase inhibitor 3	GI_38076060-S	1398	2.38	**0.000**
Fis1	fission 1 (mitochondrial outer membrane) homolog (yeast)	scl0004104.1_48-S	726	2.37	**0.000**
Kif23	kinesin family member 23	scl35732.23.1_51-S	1699	2.37	**0.000**
MSV	Murine sarcoma virus	9626100_224-S	8691	2.37	**0.000**

Sdf2l1	stromal cell-derived factor 2-like 1	scl48713.2.9_29-S	434	2.37	**0.000**
Tsc22d1	TSC22 domain family, member 1	scl000333.1_26-S	305	2.37	**0.000**
Cdkn2d	cyclin-dependent kinase inhibitor 2D (p19, inhibits CDK4)	scl36146.3_205-S	752	2.36	**0.000**
**Gcg**	glucagon	scl19223.5.1_16-S	8010	2.36	**0.000**
Lig1	ligase I, DNA, ATP-dependent	scl33069.30.1_21-S	913	2.36	**0.000**
NA	similar to RIKEN cDNA 2610524H06 gene	GI_38080728-S	255	2.36	**0.000**
Eif5a	eukaryotic translation initiation factor 5A	scl00276770.1_2-S	563	2.35	**0.000**
Eno1	enolase 1, alpha non-neuron	scl013806.12_49-S	1216	2.35	**0.000**
Fen1	flap structure specific endonuclease 1	scl014156.1_86-S	999	2.35	**0.000**
Spbc24	spindle pole body component 24 homolog (S. cerevisiae)	scl067629.2_23-S	802	2.35	**0.000**
Ppa1	pyrophosphatase (inorganic) 1	scl38868.13.1_10-S	3144	2.34	**0.000**
**Scg2**	secretogranin II	scl0020254.2_71-S	12526	2.34	**0.000**
Smc2l1	SMC2 structural maintenance of chromosomes 2-like 1 (yeast)	scl25441.25_49-S	1100	2.34	**0.000**
Gdi1	guanosine diphosphate (GDP) dissociation inhibitor 1	scl54834.6_26-S	270	2.33	**0.000**
Gpi1	glucose phosphate isomerase 1	scl0014751.2_19-S	239	2.33	**0.000**
Rpl22l1	ribosomal protein L22 like 1	scl068028.3_20-S	583	2.33	**0.000**
Tgfb1	transforming growth factor, beta 1	scl32920.6.1_10-S	201	2.33	**0.000**
Hist1h4f	histone 1, H4f	scl00319157.1_0-S	424	2.32	**0.000**
Atp6a1	ATPase, H+ transporting, lysosomal	GI_20892558-S	348	2.32	**0.000**
NA	C920004C08Rik	scl00319365.1_77-S	6147	2.31	**0.000**
Psmb3	proteasome (prosome, macropain) subunit, beta type 3	GI_31981321-S	1245	2.31	**0.000**
Hnrpa2b1	heterogeneous nuclear ribonucleoprotein A2/B1	scl0001252.1_55-S	773	2.30	**0.000**
Srm	spermidine synthase	scl0002839.1_0-S	362	2.30	**0.000**
Tmpo	thymopoietin	scl0003903.1_2-S	688	2.30	**0.000**
Hsp90ab1	heat shock protein 90kDa alpha (cytosolic), class B member 1	scl0015516.2_144-S	1619	2.29	**0.000**
Ndufs2	NADH dehydrogenase (ubiquinone) Fe-S protein 2	scl000941.1_0-S	833	2.29	**0.000**
Lgals3bp	lectin, galactoside-binding, soluble, 3 binding protein	scl39273.6_263-S	1357	2.28	**0.000**
NA	similar to Protein translation factor SUI1 homolog	GI_38076453-S	797	2.28	**0.000**
Rbmx	RNA binding motif protein, X chromosome	scl0019655.2_139-S	428	2.28	**0.000**
Tnfrsf22	tumor necrosis factor receptor superfamily, member 22	scl0319679.1_12-S	438	2.28	**0.000**
E2f2	E2F transcription factor 2	scl00242705.1_180-S	384	2.27	**0.000**
Mki67	antigen identified by monoclonal antibody Ki 67	scl30548.2_0-S	248	2.27	**0.000**
**Sst**	somatostatin	scl48628.2.9_30-S	1092	2.27	**0.005**
Tpm3	tropomyosin 3, gamma	scl059069.6_0-S	286	2.27	**0.000**
Zfp276	zinc finger protein (C2H2 type) 276	scl15331.1.1_254-S	545	2.27	**0.000**
Atp6ap2	ATPase, H+ transporting, lysosomal accessory protein 2	scl0002902.1_39-S	769	2.26	**0.000**
Ccna2	cyclin A2	scl22236.8_4-S	295	2.26	**0.000**

NA	similar to protein tyrosine phosphatase 4a1	GI_38085321-S	460	2.26	**0.000**
NA	E130306D19Rik	scl24403.4_53-S	341	2.26	**0.000**
Acot7	acyl-CoA thioesterase 7	scl24656.12.1_25-S	1694	2.25	**0.000**
Tfrc	transferrin receptor	scl49235.18_0-S	1040	2.25	**0.000**
Hist1h3e	histone 1, H3e	scl0319151.1_287-S	312	2.24	**0.000**
Pbk	PDZ binding kinase	scl052033.7_30-S	1163	2.24	**0.000**
Sqle	squalene epoxidase	scl0002494.1_23-S	242	2.24	**0.000**
Top2a	topoisomerase (DNA) II alpha	ri|2810021D13|ZX00034J13|AK028218|2851-S	308	2.24	**0.000**
Gdi1	guanosine diphosphate (GDP) dissociation inhibitor 1	scl014567.8_50-S	281	2.23	**0.000**
Spbc25	spindle pole body component 25 homolog (S. cerevisiae)	scl19174.6.1_6-S	5254	2.23	**0.000**
Clcn4-2	chloride channel 4-2	scl012727.1_314-S	336	2.22	**0.000**
NA	similar to protein 40kD (LOC380682)	GI_38091007-S	3444	2.22	**0.000**
Spnb1	spectrin beta 1	scl0020741.1_267-S	389	2.22	**0.000**
Cd24a	CD24a antigen	scl012484.2_55-S	687	2.21	**0.000**
D17H6S56E-5	DNA segment, Chr 17, human D6S56E 5	scl50002.3_113-S	830	2.21	**0.000**
Kifc1	kinesin family member C1	scl0094116.1_13-S	590	2.21	**0.000**
Metap2	methionine aminopeptidase 2	scl0056307.2_142-S	458	2.21	**0.000**
Mtch2	mitochondrial carrier homolog 2 (C. elegans)	scl0056428.2_17-S	598	2.21	**0.000**
Tnfrsf11b	tumor necrosis factor receptor superfamily, member 11b (osteoprotegerin)	scl47198.5_117-S	1040	2.21	**0.000**
Wdhd1	WD repeat and HMG-box DNA binding protein 1	scl45649.23.1_18-S	268	2.21	**0.000**
Sep15	selenoprotein	scl0002080.1_55-S	1068	2.20	**0.000**
1110002E23Rik	RIKEN cDNA 1110002E23 gene	ri|1110002E23|R000015G04|AK003291|514-S	1183	2.20	**0.000**
Crip1	cysteine-rich protein 1 (intestinal)	scl012925.2_22-S	609	2.20	**0.000**
Fkbp2	FK506 binding protein 2	scl014227.3_23-S	4653	2.20	**0.000**
Hist1h3d	histone1, H3d	scl00319149.2_275-S	318	2.20	**0.000**
NA	5830431N17Rik	scl076036.1_45-S	262	2.19	**0.000**
SV40 TAg	Large T specific_1733	SV40_large_T_Ag_specific-S	460	2.19	**0.000**
Prc1	protein regulator of cytokinesis 1	scl32477.13_570-S	3156	2.19	**0.000**
Snrpa	small nuclear ribonucleoprotein polypeptide A	scl0053607.2_0-S	250	2.19	**0.000**
Ube2c	ubiquitin-conjugating enzyme E2C	scl068612.6_90-S	251	2.19	**0.000**
Cox7a2	cytochrome c oxidase, subunit VIIa 2	scl35560.5_28-S	1581	2.18	**0.000**
NA	similar to RIKEN cDNA 2610524H06 gene	GI_38049380-S	261	2.18	**0.000**
NA	4632404B13Rik	ri|4632404B13|PX00012K06|AK028495|2689-S	547	2.18	**0.000**

Ndufa5	NADH dehydrogenase (ubiquinone) 1 alpha subcomplex, 5	scl0068202.1_15-S	3590	2.18	**0.000**
Ngfr	nerve growth factor receptor (TNFR superfamily, member 16)	scl39687.7_423-S	628	2.18	**0.000**
Rfc2	replication factor C (activator 1) 2	scl0004059.1_11-S	398	2.18	**0.000**
Sep-15	selenoprotein	scl22501.2_29-S	684	2.17	**0.000**
NA	similar to Cytochrome c, somatic	GI_38079525-S	6561	2.17	**0.000**
NA	NA	scl000048.1_50 _REVCOMP-S	946	2.17	**0.000**
Eif5a	NA	scl40009.3.137_12-S	607	2.17	**0.000**
Sfrs5	splicing factor, arginine/serine-rich 5 (SRp40, HRS)	scl0002293.1_7-S	683	2.17	**0.000**
Atp6ap2	ATPase, H+ transporting, lysosomal accessory protein 2	scl0002891.1_24-S	996	2.16	**0.000**
NA	similar to Mitochondrial import inner membrane translocase subunit TIM23	GI_38090481-S	773	2.16	**0.000**
Srprb	signal recognition particle receptor, B subunit	scl020818.5_51-S	324	2.16	**0.000**
Hist1h2bf	histone 1, H2bf	scl0319180.1_4-S	641	2.15	**0.000**
Hist1h4m	histone 1, H4m	GI_30089707-S	542	2.15	**0.000**
Hmgn2	high mobility group nucleosomal binding domain 2	scl015331.1_246-S	1084	2.15	**0.000**
Nov	nephroblastoma overexpressed gene	scl47911.5_100-S	4338	2.15	**0.000**
Nt5c3l	5'-nucleotidase, cytosolic III-like	scl0001342.1_29-S	533	2.15	**0.000**
Mt3	metallothionein 3	scl017751.1_8-S	535	2.14	**0.000**
Nt5c3l	5'-nucleotidase, cytosolic III-like	scl0001518.1_3-S	198	2.14	**0.000**
Pak3	p21 (CDKN1A)-activated kinase 3	scl018481.14_0-S	1747	2.14	**0.000**
Rab6	RAB6, member RAS oncogene family	scl00072.1_32-S	585	2.14	**0.000**
Tmem49	transmembrane protein 49	scl0001344.1_14-S	201	2.14	**0.000**
Uhrf1	ubiquitin-like, containing PHD and RING finger domains, 1	scl018140.7_263-S	2220	2.14	**0.000**
Cckbr	cholecystokinin B receptor	scl012426.5_191-S	1858	2.13	**0.000**
Cdc6	cell division cycle 6 homolog (S. cerevisiae)	scl0023834.1_74-S	526	2.13	**0.000**
NA	2610035D17Rik	scl39368.13_542-S	395	2.13	**0.000**
Rgs17	regulator of G-protein signaling 17	scl38240.6_287-S	594	2.13	**0.000**
Dynll1	dynein light chain LC8-type 1	scl056455.3_23-S	450	2.12	**0.000**
Luzp5	leucine zipper protein 5	scl076044.3_63-S	510	2.12	**0.000**
NA	similar to NADH-ubiquinone oxidoreductase ESSS subunit, mitochondrial precursor	GI_38083052-S	1642	2.12	**0.000**
Atp6v0d1	ATPase, H+ transporting, lysosomal V0 subunit D1	scl000737.1_18-S	373	2.11	**0.000**
Birc5	baculoviral IAP repeat-containing 5	scl0011799.1_42-S	370	2.11	**0.000**
Emd	emerin	scl0002880.1_15-S	411	2.11	**0.000**

NA	similar to Mitochondrial import inner membrane translocase subunit TIM23	GI_38074106-S	804	2.11	**0.000**
Sfrs10	splicing factor, arginine/serine-rich 10 (transformer 2 homolog, Drosophila)	scl0001867.1_58-S	453	2.11	**0.000**
Tm9sf2	transmembrane 9 superfamily member 2	scl000394.1_10-S	196	2.11	**0.000**
Yme1l1	YME1-like 1 (S. cerevisiae)	scl0003089.1_75-S	207	2.11	**0.000**
0610007L01Rik	RIKEN cDNA 0610007L01 gene	ri|0610007L01|R000001M10|AK002297|1310-S	1068	2.10	**0.000**
2010317E24Rik	RIKEN cDNA 2010317E24 gene	scl072080.6_214-S	477	2.10	**0.000**
Aspm	asp (abnormal spindle)-like, microcephaly associated (Drosophila)	scl17394.26.1_66-S	320	2.10	**0.000**
Cxcl12	chemokine (C-X-C motif) ligand 12	scl0001073.1_120-S	471	2.10	**0.000**
Fndc3a	fibronectin type III domain containing 3a	scl00319448.2_96-S	295	2.10	**0.000**
Hirip3	HIRA interacting protein 3	scl32046.5.1_83-S	886	2.10	**0.000**
Hspa5	heat shock 70kD protein 5 (glucose-regulated protein)	scl014828.8_22-S	1578	2.10	**0.000**
Cmas	cytidine monophospho-N-acetylneuraminic acid synthetase	scl29381.10.1_63-S	1554	2.09	**0.000**
Hnrpa3	heterogeneous nuclear ribonucleoprotein A3	scl0229279.5_20-S	500	2.09	**0.000**
Kdelr2	KDEL (Lys-Asp-Glu-Leu) endoplasmic reticulum protein retention receptor 2	scl27015.4.1_35-S	468	2.09	**0.000**
NA	1810035L17Rik	scl00380773.1_71-S	1289	2.09	**0.000**
Tcf19	transcription factor 19	scl49984.4.1_114-S	826	2.09	**0.000**
Tspan3	tetraspanin 3	scl0003580.1_5-S	836	2.09	**0.000**
Gnas	GNAS (guanine nucleotide binding protein, alpha stimulating) complex locus	scl0003148.1_1881-S	403	2.08	**0.000**
H13	histocompatibility 13	scl0003277.1_55-S	317	2.08	**0.000**
Mat2a	methionine adenosyltransferase II, alpha	scl00232087.1_95-S	399	2.08	**0.000**
Pcmt1	protein-L-isoaspartate (D-aspartate) O-methyltransferase 1	scl0003838.1_10-S	424	2.08	**0.000**
Ttc9b	tetratricopeptide repeat domain 9B	scl073032.2_11-S	229	2.08	**0.000**
Ywhaq	tyrosine 3-monooxygenase/tryptophan 5-monooxygenase activation protein, theta polypeptide	scl022630.1_91-S	1645	2.08	**0.000**
Gdi1	guanosine diphosphate (GDP) dissociation inhibitor 1	scl0014567.2_90-S	211	2.07	**0.000**
Hist1h2bj	histone 1, H2bj	scl0319183.1_124-S	402	2.07	**0.000**
Ifi27	interferon, alpha-inducible protein 27	scl0076933.2_252-S	294	2.07	**0.000**
Mlf2	myeloid leukemia factor 2	scl29514.8.22_6-S	532	2.07	**0.000**
Tmed3	transmembrane emp24 domain containing 3	scl35515.3_6-S	1149	2.07	**0.000**
Tmem32	transmembrane protein 32	scl54227.4_188-S	423	2.07	**0.000**
2310005N03Rik	RIKEN cDNA 2310005N03 gene	scl066359.2_17-S	994	2.06	**0.000**
9030625A04Rik	RIKEN cDNA 9030625A04 gene	scl45283.8.141_93-S	326	2.06	**0.000**

Atp6v0e2	ATPase, H+ transporting, lysosomal V0 subunit E2	scl0001036.1_14-S	455	2.06	**0.000**
Birc5	baculoviral IAP repeat-containing 5	scl011799.1_11-S	689	2.06	**0.000**
Calu	calumenin	scl0001217.1_1480-S	399	2.06	**0.000**
Hist1h2bc	histone 1, H2bc	scl44991.2.1_21-S	1575	2.06	**0.000**
Nudt19	nudix (nucleoside diphosphate linked moiety X)-type motif 19	scl31469.3.28_7-S	738	2.06	**0.000**
1500041J02Rik	RIKEN cDNA 1500041J02 gene	scl067876.4_28-S	654	2.05	**0.000**
2810417H13Rik	RIKEN cDNA 2810417H13 gene	scl36799.4_9-S	527	2.05	**0.000**
Hook1	hook homolog 1 (Drosophila)	scl0077963.1_19-S	574	2.05	**0.000**
Ifitm2	interferon induced transmembrane protein 2	scl080876.2_10-S	1152	2.05	**0.000**
NA	similar to RIKEN cDNA 2610524H06 gene	GI_38076908-S	205	2.05	**0.000**
Asns	asparagine synthetase	scl0001016.1_199-S	394	2.04	**0.000**
Hist1h2bm	histone 1, H2bm	scl0319186.1_75-S	488	2.04	**0.000**
Hist1h3b	histone 1, H3b	scl0319150.1_257-S	284	2.04	**0.000**
Mcm6	minichromosome maintenance deficient 6 (MIS5 homolog, S. pombe) (S. cerevisiae)	scl000980.1_16-S	645	2.04	**0.000**
NA	similar to Sid393p	GI_38085765-S	794	2.04	**0.000**
NA	1810026B05Rik	scl0069170.1_29-S	1093	2.04	**0.000**
Oact1	O-acyltransferase (membrane bound) domain containing 1	scl0218121.14_31-S	710	2.04	**0.000**
Pole	polymerase (DNA directed), epsilon	scl27442.47.1_8-S	369	2.04	**0.000**
2900010J23Rik	RIKEN cDNA 2900010J23 gene	scl19447.7.1_15-S	879	2.03	**0.000**
Incenp	inner centromere protein	scl52764.18.1_16-S	425	2.03	**0.000**
Neil3	nei like 3 (E. coli)	ri|A630055A13|PX00146D18|AK042059|2077-S	247	2.03	**0.000**
Sfrs10	splicing factor, arginine/serine-rich 10 (transformer 2 homolog, Drosophila)	scl020462.9_41-S	358	2.03	**0.000**
Tyms	thymidylate synthase	scl0004133.1_1-S	356	2.03	**0.000**
Dut	deoxyuridine triphosphatase	scl20342.7_47-S	365	2.02	**0.000**
Kifc1	kinesin family member C1	scl0016580.1_17-S	360	2.02	**0.000**
Mrps6	mitochondrial ribosomal protein S6	scl0121022.3_149-S	451	2.02	**0.000**
NA	RIKEN cDNA 1100001F19 gene	GI_20878323-S	477	2.02	**0.000**
Smc1l1	SMC (structural maintenance of chromosomes 1)-like 1 (S. cerevisiae)	scl54546.18_0-S	206	2.02	**0.000**
Taf10	TAF10 RNA polymerase II, TATA box binding protein (TBP)-associated factor	scl024075.4_0-S	651	2.02	**0.000**
Arf5	ADP-ribosylation factor 5	scl011844.1_12-S	506	2.01	**0.000**
BC031181	cDNA sequence BC031181	ri|5730496F02|PX00644L03|AK077642|980-S	3503	2.01	**0.000**

Dnm1l	dynamin 1-like	scl0074006.2_261-S	300	2.01	**0.000**
Eif4a2	eukaryotic translation initiation factor 4A2	scl0001779.1_17-S	2039	2.01	**0.000**
Pdzd11	PDZ domain containing 11	scl0002903.1_3-S	494	2.01	**0.000**
Pfkp	phosphofructokinase, platelet	scl0003623.1_49-S	556	2.01	**0.001**

[1] neuroendocrine protein genes are shown in bold; [2] p values < 0.05 are shown in bold

Gene[1]	Description	Probe set	Expression (mean) with TAg siRNA	Fold up-regulation +siRNA	P value[2]
Hrb2	HIV-1 Rev binding protein 2	ri|B230208M22|PX00069D07|AK045526|1152-S	16035	177.15	**0.000**
1810074P20Rik	RIKEN cDNA 1810074P20 gene	scl24492.20.1_105-S	16491	73.45	**0.000**
Slc7a3	solute carrier family 7 (cationic amino acid transporter, y+ system), member 3	scl53996.13.1_171-S	3475	9.17	**0.000**
Trib3	tribbles homolog 3 (Drosophila)	scl18503.5_29-S	1481	8.49	**0.022**
Crebbp	CREB binding protein	scl48815.9.1_11-S	531	5.22	**0.000**
Cacna2d1	calcium channel, voltage-dependent, alpha2/delta subunit 1	scl012293.44_74-S	1535	4.93	**0.000**
Nme7	non-metastatic cells 7, protein expressed in	scl000061.1_90_REVCOMP-S	513	4.44	**0.003**
Bat2d	BAT2 domain containing 1	ri|5730577G12|PX00093O15|AK030766|3541-S	540	4.21	**0.000**
Eif4ebp1	eukaryotic translation initiation factor 4E binding protein 1	scl013685.3_18-S	1148	4.03	**0.050**
Ddx6	DEAD (Asp-Glu-Ala-Asp) box polypeptide 6	scl0003527.1_242-S	1452	4.00	**0.000**
Slc6a9	solute carrier family 6 (neurotransmitter transporter, glycine), member 9	scl0014664.2_299-S	661	3.69	0.063
NA	2210401K01Rik	scl072289.1_141-S	507	3.55	**0.003**
Cox6a2	cytochrome c oxidase, subunit VI a, polypeptide 2	scl30620.3.1_5-S	350	3.52	**0.003**
Txnip	thioredoxin interacting protein	scl22856.7_28-S	1743	3.51	**0.000**
Hyal2	hyaluronoglucosaminidase 2	scl0015587.1_282-S	890	3.49	**0.000**
Acox2	acyl-Coenzyme A oxidase 2, branched chain	scl45913.15.1_122-S	424	3.46	**0.038**
Ankrd12	ankyrin repeat domain 12	ri|B230387C07|PX00161B10|AK046455|1022-S	571	3.45	**0.000**
NA	similar to hypothetical protein ORF-1137	GI_38080768-S	352	3.39	**0.008**
Itm2c	integral membrane protein 2C	scl17725.6_585-S	6741	3.35	**0.000**
NA	NA	scl0227620.1_20-S	603	3.26	**0.000**
Bat2d	BAT2 domain containing 1	ri|5730594E01|PX00093G24|AK077748|1826-S	320	3.22	**0.000**
NA	similar to hypothetical protein ORF-1137	GI_38081436-S	657	3.18	**0.045**
Ankrd11	ankyrin repeat domain 11	ri|B230386D16|PX00161H18|AK046451|1121-S	324	3.16	**0.000**
Cd59a	CD59a antigen	scl20534.5_360-S	1427	3.15	**0.000**
Klf3	Kruppel-like factor 3 (basic)	scl0016599.2_295-S	1689	3.14	**0.000**
Eif2c2	eukaryotic translation initiation factor 2C, 2	scl0239528.1_91-S	399	3.09	**0.000**
D16Bwg1494e	DNA segment, Chr 16, Brigham & Women's Genetics 1494 expressed	scl0224019.10_293-S	701	3.08	**0.000**

Tff3	trefoil factor 3, intestinal	scl50085.4.1_101-S	968	3.06	**0.000**
Spon2	spondin 2, extracellular matrix protein	scl26719.6_100-S	4758	3.02	**0.000**
Ppargc1a	peroxisome proliferative activated receptor, gamma, coactivator 1 alpha	scl26595.14.1_90-S	1002	3.01	**0.000**
Syne1	synaptic nuclear envelope 1	ri|4732422E11|PX00050F15|AK028641|2872-S	271	2.99	**0.013**
Zbtb7a	zinc finger and BTB domain containing 7a	scl0016969.1_242-S	381	2.97	**0.000**
Aldh6a1	aldehyde dehydrogenase family 6, subfamily A1	scl42244.15_187-S	1069	2.94	**0.000**
NA	NA	mtDNA_ND6-S	292	2.90	**0.000**
Cpd	carboxypeptidase D	scl39908.21.1_34-S	393	2.88	**0.000**
Hbp1	high mobility group box transcription factor 1	scl0002413.1_735-S	545	2.86	**0.000**
NA	similar to Ab2-162	GI_38080226-S	5648	2.86	**0.000**
NA	ORF1 (LOC386112)	GI_38081157-S	591	2.86	**0.029**
Btbd3	BTB (POZ) domain containing 3	scl00228662.1_52-S	418	2.81	0.091
Phf10	PHD finger protein 10	scl50289.12.1_79-S	2168	2.81	**0.001**
NA	1110046J11Rik	scl1329.1.1_99-S	561	2.80	**0.000**
Cln2	ceroid-lipofuscinosis, neuronal 2	scl30879.11_205-S	576	2.80	**0.000**
NA	similar to hypothetical protein ORF-1137	GI_38081412-S	283	2.77	**0.017**
Gnas	GNAS (guanine nucleotide binding protein, alpha stimulating) complex locus	scl071358.8_30-S	530	2.76	0.100
Ggnbp1	gametogenetin binding protein 1	scl0070772.1_306-S	413	2.75	**0.000**
Gstm1	glutathione S-transferase, mu 1	scl014862.2_242-S	689	2.74	**0.000**
Npc1	Niemann Pick type C1	scl51647.25_267-S	1255	2.74	**0.000**
Cpd	carboxypeptidase D	scl0012874.1_259-S	312	2.73	**0.010**
Slc16a9	solute carrier family 16 (monocarboxylic acid transporters), member 9	scl38793.6_454-S	300	2.73	**0.000**
NA	similar to RNP particle component	GI_38081272-S	974	2.71	0.099
**Calca**	calcitonin/calcitonin-related polypeptide, alpha	scl0012310.2_237-S	14398	2.70	**0.000**
Phf10	PHD finger protein 10	scl000091.1_88-S	2183	2.69	**0.001**
Retsat	retinol saturase (all trans retinol 13,14 reductase)	scl29880.12.1_23-S	2195	2.67	**0.000**
A930034L06Rik	RIKEN cDNA A930034L06 gene	scl20001.6.1_12-S	506	2.66	**0.012**
NA	RIKEN cDNA 3300001A09 gene	scl36783.2.1_280-S	5664	2.65	**0.000**
NA	6430709H04Rik	scl0319228.4_13-S	174	2.64	0.218
Stat3	signal transducer and activator of transcription 3	scl020848.1_5-S	1700	2.64	**0.000**
Ephx1	epoxide hydrolase 1, microsomal	scl15839.11.1_64-S	909	2.61	**0.000**
Slc35d3	NA	scl38150.2.1_277-S	2283	2.61	**0.000**

5730593F17Rik	RIKEN cDNA 5730593F17 gene	scl41013.18_227-S	737	2.59	**0.000**
Bbx	bobby sox homolog (Drosophila)	scl48409.23_594-S	548	2.59	**0.000**
Rbm5	RNA binding motif protein 5	scl0003447.1_0-S	699	2.57	**0.020**
Man2a1	mannosidase 2, alpha 1	scl50565.20_286-S	970	2.55	**0.000**
NA	RIKEN cDNA D330001F17 gene	scl00223658.1_328-S	2283	2.54	**0.000**
NA	RIKEN cDNA 3010027A04 gene	scl34205.11.307_7-S	402	2.52	**0.000**
Impact	imprinted and ancient	scl016210.11_31-S	707	2.52	**0.000**
Nup210	nucleoporin 210	scl28688.42_395-S	2991	2.52	**0.000**
Usp37	ubiquitin specific peptidase 37	scl0319600.1_233-S	402	2.52	**0.000**
Nupr1	nuclear protein 1	scl30680.4.1_44-S	9774	2.51	**0.015**
2310022B05Rik	RIKEN cDNA 2310022B05 gene	scl34177.5_600-S	714	2.50	**0.000**
Entpd3	ectonucleoside triphosphate diphosphohydrolase 3	scl36335.20.967_4-S	4182	2.50	**0.000**
Pdcd4	programmed cell death 4	scl52979.12_115-S	1954	2.50	**0.000**
Atf5	activating transcription factor 5	scl31408.4_131-S	1632	2.49	**0.001**
Cdkn2b	cyclin-dependent kinase inhibitor 2B (p15, inhibits CDK4)	scl24120.2_589-S	1764	2.49	**0.000**
Adamts9	Adamts9 Mus musculus a disintegrin-like and metalloprotease (reprolysin type) with thrombospondin type 1 motif, 9	scl28665.23.1_62-S	475	2.49	**0.000**
Robo1	roundabout homolog 1 (Drosophila)	scl019876.3_41-S	785	2.49	**0.000**
Ankhd1	NA	scl00108857.1_273-S	299	2.48	**0.000**
Map3k7ip2	mitogen-activated protein kinase kinase kinase 7 interacting protein 2	scl38232.7_17-S	675	2.47	**0.004**
Pcsk9	proprotein convertase subtilisin/kexin type 9	scl24041.13_589-S	398	2.47	**0.000**
Dag1	dystroglycan 1	scl013138.1_18-S	903	2.44	**0.000**
Otub2	OTU domain, ubiquitin aldehyde binding 2	scl0068149.1_319-S	307	2.43	**0.047**
1200015N20Rik	RIKEN cDNA 1200015N20 gene	scl0071721.2_141-S	477	2.42	**0.000**
Abcb4	ATP-binding cassette, sub-family B (MDR/TAP), member 4	scl26912.28.1_161-S	419	2.42	**0.000**
NA	similar to RNP particle component (LOC386124)	GI_38081206-S	336	2.42	0.090
Bat2d	BAT2 domain containing 1	ri|2900060F21|ZX00069K04|AK013732|1657-S	255	2.39	**0.016**
Ccng1	cyclin G1	scl40331.6_57-S	2346	2.38	**0.000**
Rims3	regulating synaptic membrane exocytosis 3	scl25010.7_694-S	1306	2.38	**0.000**
Tnrc6b	trinucleotide repeat containing 6b	ri|C130089M10|PX00172G11|AK048628|1520-S	262	2.38	**0.016**
Akap8l	A kinase (PRKA) anchor protein 8-like	scl054194.1_276-S	953	2.37	**0.000**
Bri3	brain protein I3	scl21206.2.1_228-S	1888	2.37	**0.000**
Dusp5	dual specificity phosphatase 5	GI_38085196-S	781	2.37	**0.000**
Srr	serine racemase	ri|B230334I23|PX00316P06|AK080830|1280-S	10002	2.37	**0.000**

Bax	Bcl2-associated X protein	scl31374.5.1_14-S	520	2.36	**0.000**
Hbp1	high mobility group box transcription factor 1	scl42553.13_222-S	497	2.36	**0.000**
6330514A18Rik	RIKEN cDNA 6330514A18 gene	scl38681.14.1_267-S	1202	2.35	**0.000**
Lasp1	LIM and SH3 protein 1	scl0016796.1_242-S	1453	2.35	**0.000**
NA	similar to matrin cyclophilin (matrin-cyp)	GI_38093603-S	508	2.35	**0.000**
Rorc	RAR-related orphan receptor gamma	scl019885.11_24-S	275	2.35	**0.000**
Slc35f3	solute carrier family 35, member F3	scl33165.11.1_4-S	405	2.35	**0.001**
Renbp	renin binding protein	scl54150.7.1_69-S	626	2.34	**0.000**
Cdkn1b	cyclin-dependent kinase inhibitor 1B (P27)	scl012576.2_22-S	285	2.33	**0.036**
Naprt1	nicotinate phosphoribosyltransferase domain containing 1	scl47060.10.1_13-S	972	2.33	**0.000**
Csad	cysteine sulfinic acid decarboxylase	scl0246277.1_158-S	661	2.32	**0.000**
Frk	fyn-related kinase	scl39029.8_154-S	598	2.31	**0.000**
NA	C630022N07Rik	scl0319360.1_291-S	856	2.31	**0.000**
Cln5	ceroid-lipofuscinosis, neuronal 5	scl46010.5_526-S	2532	2.31	**0.000**
NA	DNA segment, Chr 5, Brigham & Womens Genetics 0860 expressed	scl0052822.2_239-S	1444	2.31	**0.006**
Stat3	signal transducer and activator of transcription 3	scl0020848.1_188-S	861	2.31	**0.000**
NA	RIKEN cDNA 2310004N11 gene	scl074178.11_191-S	1075	2.30	**0.000**
Supt3h	suppressor of Ty 3 homolog (S. cerevisiae)	scl50694.13.20_68-S	1431	2.30	**0.000**
Akap9	A kinase (PRKA) anchor protein (yotiao) 9	scl0100986.1_13-S	624	2.29	**0.000**
2010315L10Rik	RIKEN cDNA 2010315L10 gene	scl33705.5.1_110-S	2138	2.28	**0.000**
Myst4	MYST histone acetyltransferase monocytic leukemia 4	scl0054169.1_231-S	368	2.28	**0.000**
Stard5	StAR-related lipid transfer (START) domain containing 5	scl00170460.2_242-S	255	2.28	**0.001**
Zfp365	zinc finger protein 365	scl37856.7_341-S	586	2.28	**0.000**
Klf6	Kruppel-like factor 6	scl023849.4_8-S	556	2.27	**0.000**
Nmb	neuromedin B	scl068039.2_3-S	700	2.27	**0.000**
Noxo1	NADPH oxidase organizer 1	scl51008.5.1_229-S	350	2.27	**0.000**
Ddit3	DNA-damage inducible transcript 3	scl38324.4.1_0-S	375	2.26	**0.002**
NA	similar to hypothetical protein ORF-1137	GI_38080884-S	283	2.26	**0.035**
Slc19a2	solute carrier family 19 (thiamine transporter), member 2	scl17283.6_608-S	667	2.26	**0.000**
Soat1	sterol O-acyltransferase 1	scl020652.2_90-S	311	2.26	**0.000**
Zbtb12	zinc finger and BTB domain containing 12	scl50802.2_363-S	446	2.25	**0.000**
Cdkn1a	cyclin-dependent kinase inhibitor 1A (P21)	scl012575.1_2-S	925	2.24	**0.000**
NA	RIKEN cDNA 2410012C07 gene	scl076484.1_294-S	4152	2.24	**0.000**
NA	5430417L22Rik	scl20442.1.104_164-S	1058	2.24	**0.000**
Csnk1g1	casein kinase 1, gamma 1	scl36794.1.1108_240-S	326	2.23	**0.000**
NA	NM_029895.3	scl21235.24_380-S	1309	2.23	**0.000**

Aplp2	amyloid beta (A4) precursor-like protein 2	scl36067.18_4-S	10188	2.22	**0.000**
Lmo4	LIM domain only 4	scl21429.6_290-S	2918	2.22	**0.000**
Mgst2	microsomal glutathione S-transferase 2	scl23214.8.1_66-S	403	2.22	**0.000**
Rxrb	retinoid X receptor beta	scl020182.9_313-S	845	2.22	**0.000**
Smarca1	SWI/SNF related, matrix associated, actin dependent regulator of chromatin, subfamily a, member 1	scl54295.26.1_21-S	867	2.22	**0.000**
Stk32a	serine/threonine kinase 32A	scl00269019.2_280-S	505	2.22	**0.000**
C530014P21Rik	RIKEN cDNA C530014P21 gene	ri|D630024O03|PX00197J13|AK085434|1298-S	247	2.21	**0.026**
NA	similar to hypothetical protein ORF-1137	GI_38081284-S	236	2.21	**0.042**
Gnpda2	Mus musculus glucosamine-6-phosphate deaminase 2	scl0067980.2_203-S	427	2.21	**0.000**
NA	RIKEN cDNA 4933421G18 gene	scl071175.1_21-S	907	2.21	**0.000**
Pdcd4	programmed cell death 4	scl0018569.1_4-S	1017	2.21	**0.000**
Ppm1l	protein phosphatase 1 (formerly 2C)-like	scl23092.5_223-S	259	2.21	**0.000**
Fbxo6b	F-box only protein 6b	scl23523.7.1_20-S	729	2.20	**0.000**
Grb7	growth factor receptor bound protein 7	scl40936.14_9-S	3131	2.20	**0.000**
Ppp1r3c	protein phosphatase 1, regulatory (inhibitor) subunit 3C	scl52526.2_193-S	384	2.20	**0.000**
Thsd4	thrombospondin, type I, domain containing 4	scl35747.13.1_171-S	273	2.20	**0.000**
Tmem23	transmembrane protein 23	scl52550.23_145-S	445	2.20	**0.000**
Gcc2	GRIP and coiled-coil domain containing 2	ri|2600014C01|ZX00060D21|AK011206|858-S	229	2.19	**0.011**
Scamp5	secretory carrier membrane protein 5	scl35792.9_485-S	3418	2.19	**0.000**
1200016E24Rik	RIKEN cDNA 1200016E24 gene	ri|4833436O22|PX00028P19|AK029441|2683-S	504	2.18	**0.000**
4931429I11Rik	RIKEN cDNA 4931429I11 gene	scl0003510.1_178-S	235	2.18	**0.000**
Wbscr16	Williams-Beuren syndrome chromosome region 16 homolog (human)	scl25953.10_428-S	390	2.18	**0.000**
2310015B20Rik	RIKEN cDNA 2310015B20 gene	scl38794.5.1_91-S	807	2.17	**0.000**
LOC213480	similar to RIKEN cDNA C920008G01 gene	GI_38076290-S	931	2.17	**0.050**
Noxo1	NADPH oxidase organizer 1	scl0001724.1_23-S	292	2.17	**0.000**
Por	P450 (cytochrome) oxidoreductase	scl018984.16_308-S	476	2.17	**0.000**
Pros1	protein S (alpha)	scl49002.15_178-S	1077	2.17	**0.000**
Gria3	glutamate receptor, ionotropic, AMPA3 (alpha 3)	scl8260.1.1_298-S	324	2.16	**0.000**
Gtf2h4	general transcription factor II H, polypeptide 4	scl49981.15.1_56-S	869	2.16	**0.000**
Llgl1	lethal giant larvae homolog 1 (Drosophila)	scl41479.21_1-S	618	2.16	**0.000**
Nfatc3	nuclear factor of activated T-cells, cytoplasmic, calcineurin-dependent 3	scl33393.12_260-S	590	2.16	**0.000**
Ypel5	yippee-like 5 (Drosophila)	scl0383295.3_18-S	873	2.16	**0.000**
Akt2	thymoma viral proto-oncogene 2	scl32891.21_547-S	342	2.15	**0.000**

Lrrc35	leucine rich repeat containing 35	scl35981.11_448-S	1053	2.15	**0.000**
Rbpms	RNA binding protein gene with multiple splicing	scl34939.16_10-S	266	2.15	**0.000**
Slc1a4	solute carrier family 1 (glutamate/neutral amino acid transporter), member 4	scl40472.8_474-S	2086	2.15	**0.004**
Snx30	sorting nexin family member 30	scl00209131.1_196-S	266	2.15	**0.000**
Zzef1	zinc finger, ZZ-type with EF hand domain 1	scl0195018.6_13-S	400	2.15	**0.000**
3300001P08Rik	RIKEN cDNA 3300001P08 gene	scl067684.2_37-S	1533	2.14	**0.003**
5830434P21Rik	RIKEN cDNA 5830434P21 gene	scl21064.31_2-S	1977	2.14	**0.000**
Il6ra	interleukin 6 receptor, alpha	ri|9530096I01|PX00114B19|AK020663|941-S	246	2.14	**0.000**
Mll1	myeloid/lymphoid or mixed-lineage leukemia 1	scl35942.36_189-S	1956	2.14	**0.000**
Sltm	SAFB-like, transcription modulator	scl066660.3_29-S	698	2.14	**0.000**
Tmem51	transmembrane protein 51	scl0214359.1_299-S	754	2.14	**0.000**
Bcl11a	B-cell CLL/lymphoma 11A (zinc finger protein)	scl0001352.1_150-S	415	2.13	**0.000**
D0H4S114	DNA segment, human D4S114	scl51556.7_209-S	635	2.13	**0.000**
Eif3s1	eukaryotic translation initiation factor 3, subunit 1 alpha	ri|2700079K05|ZX00082L10|AK019219|263-S	344	2.13	**0.000**
NA	6230400G14Rik	scl28675.1.1_200-S	559	2.13	**0.000**
NA	NA	scl000034.1_162_REVCOMP-S	237	2.12	**0.000**
NA	RIKEN cDNA 2810455F06 gene	scl0227570.7_253-S	874	2.12	**0.000**
NA	1110003O08Rik	scl34240.1.561_330-S	719	2.12	**0.000**
Pdpk1	3-phosphoinositide dependent protein kinase-1	scl50221.17.1_145-S	596	2.12	**0.000**
2810037C14Rik	RIKEN cDNA 2810037C14 gene	scl067211.6_180-S	740	2.11	**0.000**
Atbf1	AT motif binding factor 1	scl011906.16_208-S	892	2.11	**0.000**
Chac1	ChaC, cation transport regulator-like 1 (E. coli)	scl20421.3_574-S	856	2.11	0.173
Acadsb	acyl-Coenzyme A dehydrogenase, short/branched chain	scl0066885.2_329-S	2038	2.10	**0.000**
Dag1	dystroglycan 1	scl35355.5_339-S	807	2.10	**0.000**
NA	similar to RNP particle component (LOC386169)	GI_38081258-S	237	2.10	**0.008**
NA	RIKEN cDNA 5830416A07 gene	scl076014.3_8-S	663	2.10	**0.000**
Sema5a	sema domain, seven thrombospondin repeats (type 1 and type 1-like), transmembrane domain (TM) and short cytoplasmic domain, (semaphorin) 5A	scl0020356.1_230-S	386	2.10	**0.000**
Tsta3	tissue specific transplantation antigen P35B	scl47056.10.1_17-S	1403	2.10	**0.000**
Irf2	interferon regulatory factor 2	scl33841.11_453-S	1115	2.09	**0.000**
NA	6330509M05Rik	scl7210.1.1_253-S	1308	2.09	**0.000**
2600005C20Rik	RIKEN cDNA 2600005C20 gene	scl072462.6_25-S	327	2.08	**0.000**
Blcap	bladder cancer associated protein homolog (human)	scl18420.2_47-S	624	2.08	**0.000**

Lrrn1	leucine rich repeat protein 1, neuronal	scl29678.2_366-S	663	2.08	**0.000**
NA	RIKEN cDNA 5830407P18 gene	scl0078818.1_318-S	239	2.08	**0.039**
Smarcd2	SWI/SNF related, matrix associated, actin dependent regulator of chromatin, subfamily d, member 2	scl083796.1_300-S	1432	2.07	**0.000**
5730509C05Rik	RIKEN cDNA 5730509C05 gene	scl40925.4.3563_8-S	407	2.06	0.105
Ap1b1	adaptor protein complex AP-1, beta 1 subunit	scl011764.20_169-S	2376	2.06	**0.000**
Mrps34	mitochondrial ribosomal protein S34	scl50998.1_28-S	1874	2.06	**0.000**
NA	RIKEN cDNA 1200003E16 gene	scl066860.1_6-S	665	2.06	**0.000**
NA	A830080H07Rik	scl37344.1.417_0-S	1036	2.06	**0.000**
NA	cDNA sequence BC003940	scl39231.4_382-S	1628	2.06	**0.000**
Prkrir	protein-kinase, interferon-inducible double stranded RNA dependent inhibitor, repressor of (P58 repressor)	scl0072981.1_191-S	561	2.06	**0.000**
Slc25a28	solute carrier family 25, member 28	scl52458.4_47-S	1059	2.06	**0.000**
Slc7a14	solute carrier family 7 (cationic amino acid transporter, y+ system), member 14	scl00241919.1_47-S	296	2.06	**0.000**
Tcof1	Treacher Collins Franceschetti syndrome 1, homolog	scl0021453.2_208-S	608	2.06	**0.000**
1810027O10Rik	RIKEN cDNA 1810027O10 gene	scl41358.1_245-S	6304	2.04	**0.000**
NA	similar to KIAA1731 protein	GI_38090253-S	299	2.04	**0.000**
Mtap2	microtubule-associated protein 2	ri|A330078K03|PX00133N11|AK079618|3541-S	213	2.04	0.060
NA	2310040A07Rik	scl24418.2_44-S	566	2.04	**0.000**
NA	1810027I20Rik	scl26483.7_409-S	291	2.04	**0.000**
NA	E330039G21Rik	scl40721.18.1_28-S	332	2.04	**0.000**
Pacs1	phosphofurin acidic cluster sorting protein 1	scl53489.23_479-S	634	2.04	**0.000**
Odz3	odd Oz/ten-m homolog 3 (Drosophila)	scl0023965.1_142-S	462	2.04	**0.000**
Pclo	piccolo (presynaptic cytomatrix protein)	scl0026875.1_310-S	755	2.04	**0.000**
Tjp1	tight junction protein 1	scl31237.29_375-S	326	2.04	**0.031**
Mtap1b	microtubule-associated protein 1 B	scl017755.1_80-S	481	2.03	**0.017**
NA	similar to hypothetical protein ORF-1137	GI_38081151-S	218	2.03	**0.008**
Gnai1	guanine nucleotide binding protein, alpha inhibiting 1	scl014677.2_17-S	282	2.03	**0.000**
Ptprd	protein tyrosine phosphatase, receptor type, D	scl070222.2_44-S	697	2.03	**0.000**
Trp53inp1	transformation related protein 53 inducible nuclear protein 1	scl25678.6_114-S	472	2.03	**0.000**
Zzz3	zinc finger, ZZ domain containing 3	scl22447.5.1_50-S	201	2.03	**0.000**
C530044N13Rik	RIKEN cDNA C530044N13 gene	scl48750.6_548-S	1159	2.02	**0.000**
Fvt1	follicular lymphoma variant translocation 1	scl16414.9.494_144-S	523	2.02	**0.000**
Mt2	metallothionein 2	scl33493.3.1_90-S	678	2.02	**0.000**

NA	hypothetical protein A730098P15	scl0209012.1_266-S	262	2.02	**0.004**
Sharpin	SHANK-associated RH domain interacting protein	scl47044.6.24_75-S	1569	2.02	**0.000**
9930021J03Rik	RIKEN cDNA 9930021J03 gene	scl52589.1_716-S	307	2.01	**0.000**
Adra2a	adrenergic receptor, alpha 2a	scl52981.3.767_203-S	1185	2.01	**0.000**
Camk4	calcium/calmodulin-dependent protein kinase IV	scl15233.1.1_225-S	297	2.01	**0.000**
Ddit4l	DNA-damage-inducible transcript 4-like	scl22560.5_561-S	1451	2.01	**0.000**
Flcn	folliculin	scl40154.14_234-S	1017	2.01	**0.000**
Mdm2	transformed mouse 3T3 cell double minute 2	scl37463.11_46-S	1726	2.01	**0.000**
Pdap1	PDGFA associated protein 1	scl25784.5.1_54-S	1341	2.01	**0.003**
NA	A730017D01Rik	scl28224.2_30-S	569	2.01	**0.000**
NA	RIKEN cDNA D430042O09 gene	scl32066.25_84-S	782	2.01	**0.000**
Rbms3	RNA binding motif, single stranded interacting protein	scl35242.18_241-S	591	2.01	**0.001**
Tap2	transporter 2, ATP-binding cassette, sub-family B (MDR/TAP)	scl50827.12.1_175-S	518	2.01	**0.000**

[1] neuroendocrine protein genes are shown in bold; [2] p values < 0.05 are shown in bold
