# Supplementary material for: Expression of a Neuroendocrine Gene Signature in Gastric Tumor Cells from CEA 424-SV40 Large T Antigen-Transgenic Mice Depends on SV40 Large T Antigen
Source: PLoS One. 2012 Jan 13;7(1):e29846. doi: 10.1371/journal.pone.0029846 (PMC3258231; doi:10.1371/journal.pone.0029846)
Supplement: Table S5 — Neuroendocrine phenotype in SV40-TAg-transgenic mouse tumor models. (PDF) [file pone.0029846.s006.pdf]

Table S5: Neuroendocrine phenotype in SV40-TAg-transgenic mouse tumor models

Mouse strain	promoter (size/location relative to translational start)	tumor location	classification	evidence for classification of tumor (marker)	reference
neuroendocrine tumors					
CEA424-SV40 TAg	human *CEACAM5* (424 bp)	antrum	neuroendocrine tumor	transcriptome analysis; electron microscopy; immunohistology (CGHA); serotonin in blood	this paper
Kit-SV40 TAg	mouse *Kit*	multiple (pituitary, thyroid)	neuroendocrine tumor	immunohistology (CGHA, ENO2)	Bosse et al. 1997
Vil-Cre-ER^{T2} x LoxP-SV40 TAg	murine *Vil1* (9 kb)	colon	neuroendocrine tumor, glandular carcinoma	immunohistology (SYP)	Czeh et al. 2010
CR2-SV40 TAg	mouse *Defa2/cryptdin-2* (6.5 kb)	prostate	neuroendocrine tumor	immunohistology (CHGA, SYP)	Garabedian et al. 1998
rPB-SV40 TAg	rat *Pbsn* (-426 to +28 bp)	prostate	neuroendocrine tumor	immunohistology (SYP)	Chiaverotti et al. 2008
12T-10-SV40 TAg	rat *Pbsn* (-11,500 to +28 bp)	prostate	neuroendocrine tumor, adenocarcinoma	immunohistology (CGHA)	Masumori et al. 2001
ITF-SV40 TAg	mouse *TFF3/ITF* (-1,190 to +59)	proximal colon	neuroendocrine tumor	immunohistology (SYP)	Gum et al. 2004
RIP1-Tag2	rat *Ins2*	prostate	neuroendocrine tumor	transcriptome analysis (Gcg, Chga, Chgb, Scg2, Sct)[1]	Hanahan 1985; Djokovic et al. 2010
I-FABP$^{-1178\ to\ +28}$-SV40 TAg	rat *Fabp2/I-FABP* (-1178 to +28)	proximal colon (submucosal)	neuroendocrine tumor	electron microscopy	Kim et al. 1993
GLU-SV40 TAg	rat *Ggn* (2.1 kb)	colon, pancreas	endocrine tumor	electron microscopy; immunohistology (PYY, GGN)	Lee et al. 1992; Asa et al. 1996
Gγ/T-15-SV40 TAg	human *fetal Gγ globin* (4 kb)	prostate	neuroendocrine tumor, epithelial tumor	immunohistology (CGHA, KRT8)	Perez-Stable et al. 1997
ATP4b-SV40 TAg	murine *ATP4b* (–1,035 to +24)	stomach	neuroendocrine tumor	transcriptome analysis; electron microscopy; immunohistology (CGHA, DDC)	Syder et al. 2004
PYY-SV40 TAg	rat *PYY* (2.8 kb)	colon	endocrine tumor	immunohistology (SCT, GCG, gastrin, neurotensin, serotonin)	Upchurch et al. 1996
non-endocrine tumors					
Misiir-SV40 TAg	mouse *Amhr2/Misiir*	ovaries	carcinoma, poorly differentiated	Immunohistology (SYP, KRT8, KRT19)	Connolly et al. 2003
C3(1)-SV40 TAg	rat *Psbpc1/C3(1)* (5 kb)	mammary gland prostate	adenocarcinoma		Maroulakou et al. 1994
TRP-1-SV40 TAg	*Tyrp1* (1.4 kb)	eye	epitheliual tumor	Immunohistology (TYRP1, TYR, KRT)	Penna et al. 1998
Tyr-SV40 TAg	mouse *Tyr* (338 bp)	eye	melanoma (choroidal)	Immunohistology (S-100, gp100)	Syed et al. 1998
WAP-SV40 TAg	mouse *Wap* (1.4 kb)	mammary gland	adenocarcinoma	Immunohistology (KRT8, KRT18)	Tzeng et al. 1993; Schulze-Garg et al. 2000
SP-C-Sv40 TAg	huma *SFTPC* (3,683 to +18)	lung	adenocarcinoma		Wikenheiser et al. 1992

[1] Data obtained from http://www.ncbi.nlm.nih.gov/geo/query/acc.cgi?acc=GSE24603
