# Supplementary material for: Expression of a Neuroendocrine Gene Signature in Gastric Tumor Cells from CEA 424-SV40 Large T Antigen-Transgenic Mice Depends on SV40 Large T Antigen
Source: PLoS One. 2012 Jan 13;7(1):e29846. doi: 10.1371/journal.pone.0029846 (PMC3258231; doi:10.1371/journal.pone.0029846)
Supplement: References S1 — References for Table S5. (PDF) [file pone.0029846.s007.pdf]

Supporting References

Asa SL, Lee YC and Drucker DJ. (1996). Development of colonic and pancreatic endocrine tumours in mice expressing a glucagon-SV40 T antigen transgene. *Virchows Arch,* **427:** 595-606.

Bosse P, Bernex F, De SP, Salaun P and Panthier JJ. (1997). Multiple neuroendocrine tumours in transgenic mice induced by c-kit-SV40 T antigen fusion genes. *Oncogene,* **14:** 2661-2670.

Connolly DC, Bao R, Nikitin AY, Stephens KC, Poole TW, Hua X et al. (2003). Female mice chimeric for expression of the simian virus 40 TAg under control of the MISIIR promoter develop epithelial ovarian cancer. *Cancer Res,* **63:** 1389-1397.

Djokovic D, Trindade A, Gigante J, Badenes M, Silva L, Liu R et al. (2010). Combination of Dll4/Notch and Ephrin-B2/EphB4 targeted therapy is highly effective in disrupting tumor angiogenesis. *BMC Cancer,* **10:** 641.

Garabedian EM, Humphrey PA and Gordon JI. (1998). A transgenic mouse model of metastatic prostate cancer originating from neuroendocrine cells. *Proc Natl Acad Sci U S A,* **95:** 15382-15387.

Hanahan D. (1985). Heritable formation of pancreatic beta-cell tumours in transgenic mice expressing recombinant insulin/simian virus 40 oncogenes. *Nature,* **315:** 115-122.

Kim SH, Roth KA, Moser AR and Gordon JI. (1993). Transgenic mouse models that explore the multistep hypothesis of intestinal neoplasia. *J Cell Biol,* **123:** 877-893.

Lee YC, Asa SL and Drucker DJ. (1992). Glucagon gene 5'-flanking sequences direct expression of simian virus 40 large T antigen to the intestine, producing carcinoma of the large bowel in transgenic mice. *J Biol Chem,* **267:** 10705-10708.

Maroulakou IG, Anver M, Garrett L and Green JE. (1994). Prostate and mammary adenocarcinoma in transgenic mice carrying a rat C3(1) simian virus 40 large tumor antigen fusion gene. *Proc Natl Acad Sci U S A,* **91:** 11236-11240.

Masumori N, Thomas TZ, Chaurand P, Case T, Paul M, Kasper S et al. (2001). A probasin-large T antigen transgenic mouse line develops prostate adenocarcinoma and neuroendocrine carcinoma with metastatic potential. *Cancer Res,* **61:** 2239-2249.

Penna D, Schmidt A and Beermann F. (1998). Tumors of the retinal pigment epithelium metastasize to inguinal lymph nodes and spleen in tyrosinase-related protein 1/SV40 T antigen transgenic mice. *Oncogene,* **17:** 2601-2607.

Perez-Stable C, Altman NH, Mehta PP, Deftos LJ and Roos BA. (1997). Prostate cancer progression, metastasis, and gene expression in transgenic mice. *Cancer Res,* **57:** 900-906.

Schulze-Garg C, Lohler J, Gocht A and Deppert W. (2000). A transgenic mouse model for the ductal carcinoma in situ (DCIS) of the mammary gland. *Oncogene,* **19:** 1028-1037.

Syed NA, Windle JJ, Darjatmoko SR, Lokken JM, Steeves RA, Chappell R et al. (1998). Transgenic mice with pigmented intraocular tumors: tissue of origin and treatment. *Invest Ophthalmol Vis Sci,* **39:** 2800-2805.

Tzeng YJ, Guhl E, Graessmann M and Graessmann A. (1993). Breast cancer formation in transgenic animals induced by the whey acidic protein SV40 T antigen (WAP-SV-T) hybrid gene. *Oncogene,* **8:** 1965-1971.

Upchurch BH, Fung BP, Rindi G, Ronco A and Leiter AB. (1996). Peptide YY expression is an early event in colonic endocrine cell differentiation: evidence from normal and transgenic mice. *Development,* **122:** 1157-1163.

Wikenheiser KA, Clark JC, Linnoila RI, Stahlman MT and Whitsett JA. (1992). Simian virus 40 large T antigen directed by transcriptional elements of the human surfactant protein C gene produces pulmonary adenocarcinomas in transgenic mice. *Cancer Res,* **52:** 5342-5352.
